# Targeting autophagy using metallic nanoparticles: a promising strategy for cancer treatment

**DOI:** 10.1007/s00018-018-2973-y

**Published:** 2018-11-27

**Authors:** Marco Cordani, Álvaro Somoza

**Affiliations:** 1 Instituto Madrileño de Estudios Avanzados en Nanociencia (IMDEA Nanociencia), CNB-CSIC-IMDEA Nanociencia Associated Unit “Unidad de Nanobiotecnología”, Madrid, Spain; 2 Institute for Advanced Studies in Nanoscience (IMDEA Nanociencia), Faraday 9, Office 129, Lab 137 Ciudad Universitaria de Cantoblanco, 28049 Madrid, Spain

**Keywords:** Nanomedicine, Nanomaterials, Cancer therapy, Autophagy

## Abstract

Despite the extensive genetic and phenotypic variations present in the different tumors, they frequently share common metabolic alterations, such as autophagy. Autophagy is a self-degradative process in response to stresses by which damaged macromolecules and organelles are targeted by autophagic vesicles to lysosomes and then eliminated. It is known that autophagy dysfunctions can promote tumorigenesis and cancer development, but, interestingly, its overstimulation by cytotoxic drugs may also induce cell death and chemosensitivity. For this reason, the possibility to modulate autophagy may represent a valid therapeutic approach to treat different types of cancers and a variety of clinical trials, using autophagy modulators, are currently employed. On the other hand, recent progress in nanotechnology offers plenty of tools to fight cancer with innovative and efficient therapeutic agents by overcoming obstacles usually encountered with traditional drugs. Interestingly, nanomaterials can modulate autophagy and have been exploited as therapeutic agents against cancer. In this article, we summarize the most recent advances in the application of metallic nanostructures as potent modulators of autophagy process through multiple mechanisms, stressing their therapeutic implications in cancer diseases. For this reason, we believe that autophagy modulation with nanoparticle-based strategies would acquire clinical relevance in the near future, as a complementary therapy for the treatment of cancers and other diseases.

## Introduction

Cancer is a complex set of diseases that represent almost one-third of the leading causes of death and disability worldwide [1]. Although the tumors widely differ from their genetic and molecular basis, phenotypic manifestations and variability on the prognosis, they share common hallmarks such as self-sustained proliferative abilities, sustained angiogenesis, drastic metabolic alterations, or the capability to invade surrounding tissues and metastasize [2]. In addition, a relevant number of human tumors display dysregulation on autophagy, the essential cellular housekeeping mechanism that enables eukaryotic organisms to maintain cellular homeostasis and normal function by degrading and turning over damaged organelles and misfolded proteins [3, 4]. In tumors with enhanced autophagy, its inhibition affects tumor cell survival under metabolic and chemotherapy stresses. On the other hand, excessive autophagy induction by cytotoxic drugs or autophagy inducers may also lead to autophagic cell death. Hence, the modulation of autophagy represents a therapeutic approach for different types of cancers [5]. Traditional chemotherapeutic drugs present a variety of side effects such as low specificity, irregular distribution in tissues and organs, rapid drug clearance and biodegradation [6]. Therefore, new cancer treatments are desired, such as those derived from nanomedicine. This field of research can be defined as nanotechnology applied to human health and provides novel approaches for treating many human diseases, including cancer [7]. The majority of nanomaterials exhibit unique properties that make them useful for a variety of biotechnological applications. These properties have been exploited to create effective therapeutic and/or diagnostic tools [8, 9]. Nanomaterials can be used as cytotoxics, and/or enhancers of standard chemotherapies, as well as, drug delivery systems, reducing the side effects of conventional drugs [10, 11]. A number of nanomedicines have been assessed in clinical trials in combination with various therapeutic agents, mainly anticancer drugs, and many more are expected to be approved by the Food and Drug Administration (FDA) in the near future [12, 13]. Interestingly, several studies have reported the ability of various types of nanomaterials to exert a cytotoxic effect by modulating the autophagy process [14, 15]. Despite the risk of their inherent toxicity in immunity cells, and cardiovascular and neurological systems, nanomaterials may serve as therapeutic agents against autophagy-related diseases [14, 15]. In this review, we summarize the recent studies on the capability of nanostructures to promote cell death by autophagy overstimulation in cancerous cells. Furthermore, to better introduce the reader to this topic we have included some sections dealing with autophagy, where its mechanisms and implications are detailed, as well, as some uses of nanomaterials in nanomedicine.

## Macroautophagy

**Fig. 1:**
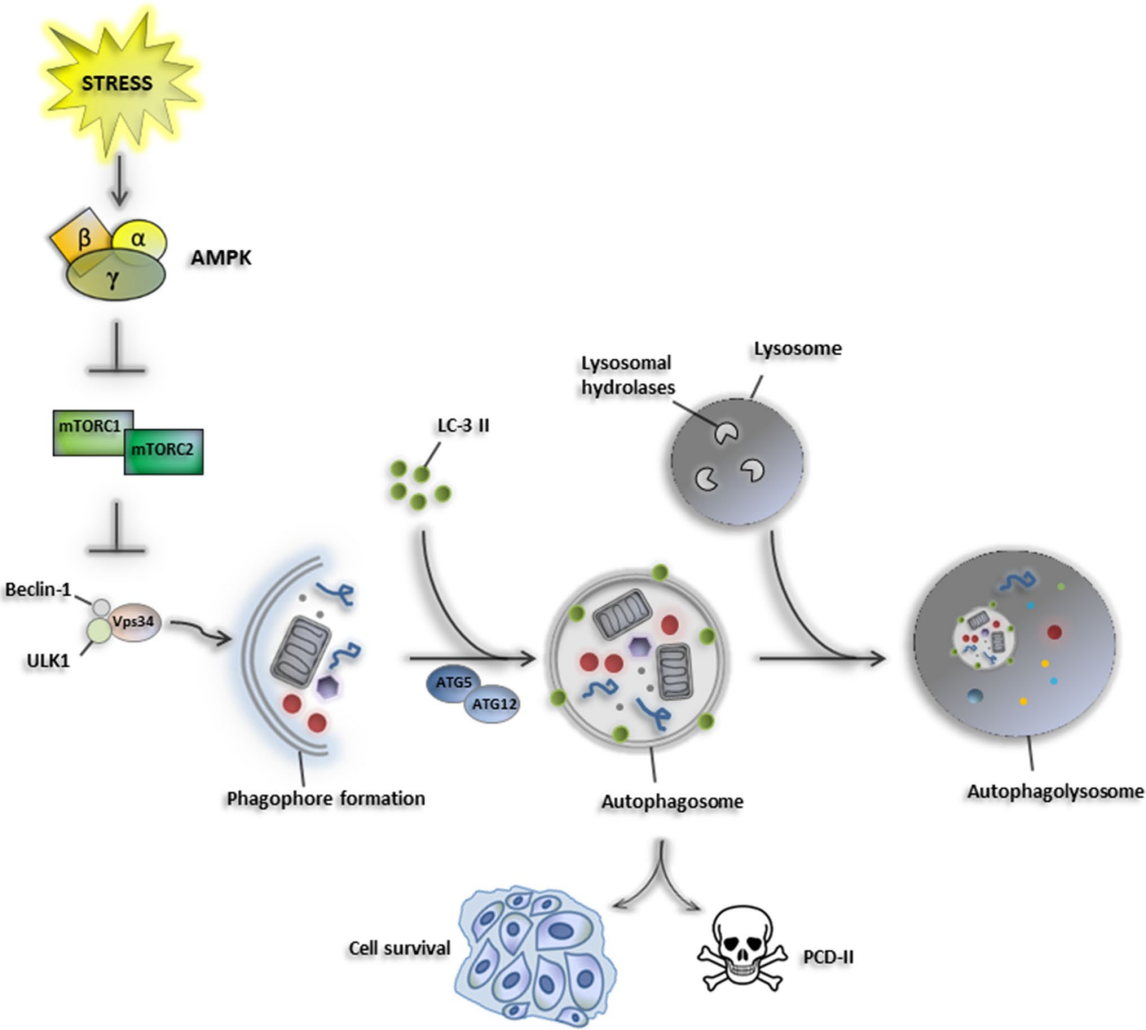
Mechanism of macroautophagy. Cellular stresses induce AMPK signaling that inhibits the anti-autophagic mTOR complex (mTORC1 and mTORC2). Consequently, Beclin-1, ULK1, and Vps34 mediate phagophore formation and autophagy initiation. Recruitment of LC-3 II into the growing phagophore is dependent on ATG5–ATG12 interaction which favors the binding of LC3B-II on both internal and external surfaces of autophagosomes, where it plays a role in both fusion of membranes with lysosomes and in selecting cargo for lysosomal degradation. Depending on the nature of the stimulus and by cellular context, autophagy acts as a pro-survival mechanism by maintaining vital cellular activities, or drives cell death-type-II, thus acting as tumor suppressor event

Macroautophagy (commonly referred to as autophagy) is an intracellular degradative process by which damaged macromolecules and organelles are targeted by autophagic vesicles to lysosomes and then eliminated. Autophagy is crucial to maintain primary biological activities during cellular stresses, such as nutrient starvation [16]. Once autophagy is activated, the cellular components are embedded into double-membrane vesicles (autophagosomes), which fuse with lysosomes to form an autophagolysosome structure to degrade its contents by lysosomal hydrolases providing a nutrient source for maintaining vital cellular activities [17]. Autophagy requires the activation of some autophagy-related genes (ATGs), which play a pivotal role in the formation of double-membrane autophagosome vesicles and the stimulation of the autophagy machinery [18, 19]. Vesicular protein sorting 34 (Vps34), belonging to the class III PI-3 kinases, has been described to interact with Beclin-1 and other autophagy-related proteins playing a critical role in autophagy initiation [19]. Importantly, the ubiquitin-like conjugation systems are necessary for the activity of specific ATG proteins [20]. In particular, the mammalian homolog of ATG8, also called LC3B, is expressed as a full-length cytosolic protein that, upon induction of autophagy, is proteolytically cleaved by ATG4, a cysteine protease, to generate LC3B-I. The carboxy-terminal glycine exposed by ATG4-dependent cleavage is then activated in an ATP-dependent manner by the E1-like ATG7 and transferred to ATG3, to generate the active isoform LC3B-II. The recruitment and integration of LC3B-II into the growing phagophore are dependent on ATG5–ATG12 interaction, favoring the binding of LC3B-II on both internal and external surfaces of autophagosomes, where it plays a role in both fusion of membranes and in selecting cargo for lysosomal degradation [21] (Fig. 1).

### AMPK and mTOR: the regulators of autophagy

The nutrient energy sensor AMP-activated protein kinase (AMPK) is the master regulator of autophagy. It inhibits mTORC1 through phosphorylation of TSC2 and Raptor in response to cellular energy cues [22, 23] and the AMPK-dependent ULK1 phosphorylation is a required step to trigger the autophagy machinery [24]. Under energetic stress, autophagy initiators unc-51-like kinase 1 (ULK1) complex promotes autophagy by targeting several downstream crucial autophagy effectors involved in the initiation of the process, such as the actin-associated motor protein myosin II and ATG9 [24]. Mammalian target of rapamycin (mTOR), a serine/threonine protein kinase with large molecular size, belongs to the phosphatidylinositol kinase-related kinase (PIKK) family and it is implicated in the regulation of multiple cellular processes including cell growth, cell cycle, cell survival, as well as autophagy. The observation that treatment with mTOR inhibitors, such as rapamycin, is sufficient to induce autophagy even in the presence of nutrients represents valid evidence for the conclusion that mTOR complex is a powerful repressor of autophagy [25]. mTOR is composed of two multiprotein enzymatic functional complexes, mTORC1 and mTORC2 [26]. Contrarily to mTORC2, mTORC1 is sensitive to the inhibition by rapamycin, and it is directly regulated by the cellular nutrient status, including growth factors and amino acid availability, playing essential roles in the regulation of protein translation and autophagy [26]. Although mTORC2 was discovered recently, it has already been demonstrated that mTORC2 plays a role in chaperone-mediated autophagy [27] and could activate autophagy via FoxO3 [28]. Interestingly, mTORC2 phosphorylates and activates Akt, which has a role in the regulation of cell proliferation, survival, metabolism, and transcription [26]. Genetic and biochemical studies demonstrated that the inhibition of ULK1 by mTOR is a crucial mechanism involved in autophagy repression [29]. Importantly, recent studies showed that mTOR can phosphorylate ULK1 on Ser757 to favor autophagy blockage [24]. Several studies indicate that wild-type p53 protein triggers autophagy in cancer cells through various mechanisms including the stimulation of AMPK, the inhibition of the mTOR (by upregulation of PTEN and TSC1), and the induction of DRAM1 [30]. The functional interplay between AMPK and wtp53 is a well-described mechanism involved in tumor suppression. Indeed, the stimulation of AMPK by energy stress promotes the phosphorylation and activation of wtp53 [31, 32]. Moreover, AMPK can increase both the activity and the stability of wtp53 through direct phosphorylation of p53 and inactivation of MDMX-mediated ubiquitination process, thus prolonging the half-life of wtp53 itself [33]. Wtp53 may, in turn, increase AMPK activity through transcriptional activation of the gene encoding the β subunit of the enzymatic complex [34] and Sestrins [35], providing positive feedback that sustains an autophagic AMPK signaling.

### Mitophagy: a specialized form of autophagy

Mitochondria are cellular organelles playing a crucial role in energy metabolism, regulation of cell signaling and apoptosis in eukaryotic cells [36]. To maintain cellular homeostasis, the cell has evolved complex systems for the quality control and clearance of mitochondria. Mitophagy is a selective form of autophagy, by which dysfunctional or damaged mitochondria are selectively targeted by autophagosomes and delivered to lysosomes to be recycled by the cell. Hence, mitophagy represents an essential quality control mechanism to ensure mitochondrial network’s integrity and functionality. Similarly to macroautophagy, mitophagy is tightly regulated by a variety of proteins controlling each phase of the process, to ensure the selective sequestration of those mitochondria that need to be eliminated, in the forming autophagosome. Efficient mitophagy occurs during some vital biological processes through Parkin-dependent and Parkin-independent pathways, and allows the damaged organelles to be targeted into the nascent autophagosome, without affecting the entire mitochondrial network [37, 38]. However, an extensive or uncontrolled mitophagy can lead to bioenergetic failure, whereas excessive mitochondrial biogenesis can generate high levels of reactive oxygen species (ROS) and promote apoptosis or cell survival depending on the type of stimulus and the cellular context [38]. Therefore, the maintenance of a balanced healthy mitochondrial population through both processes is essential for cellular function and survival [39]. Defects in mitophagy machinery are linked to most of the neurodegenerative diseases [40], to tumorigenesis, neoplastic progression and chemoresistance [41, 42]. Therefore, we hypothesize that pharmacological modulation of mitophagy could represent a potent strategy against many human diseases.

### Autophagy in cancer

Autophagy has been described to play a role in physiological processes, mammalian development and a variety of human diseases, including cancer, neurodegenerative diseases and muscular disorders [43]. Autophagy regulation is strictly interconnected with the aberrant setting of cancer cell metabolism as revealed by the fact that mTOR and AMPK pathways are both the master regulators of autophagy and the most critical sensors of the cellular energy status [44]. In particular, the mTOR complex stimulates anabolic biosynthesis for cancer cell growth and inhibits autophagy, while AMPK signaling triggers the degradation of macromolecules, including lysosomal autophagic catabolism [45]. The role of autophagy in regulating cancer cell death or survival remains highly controversial and it is likely reliant on the tumor type, the stage of neoplasia and the cellular context, as well as by metabolic context in which the cells lie [46].

Some studies support the idea that, in established tumors, constitutive autophagy may have a protective role in cancer cells by removing damaged organelles or recycling misfolded macromolecules [47]. In support of this hypothesis, several studies report that autophagy tries to fulfill the high metabolic demands of the proliferating tumor cells exposed to stressful conditions, such as nutrient deprivation, oxidative stress, hypoxia, or in response to therapy [46, 48]. Hypoxic microenvironments trigger HIF-1α-dependent and -independent autophagy, which also contributes to tumor survival [49]. Interestingly, cancers harboring activating KRAS mutations have a high basal rate of autophagy, even in growth conditions [50]. It has been reported that pharmacological and genetic inhibition of autophagy results in tumor regression in pancreatic cancer xenografts and genetic mouse models [50, 51]. Thus, by enhancing stress tolerance and providing an alternative nutrient source by which cancer cells can meet their massive nutrient and energy demands, autophagy appears to serve as a pro-survival mechanism for tumor cells.

However, it is also well established that cancer cells having uncontrolled autophagy can also undergo cell death, also called cell death-type II, likely due to excessive degradation of cellular constituents and organelles required for homeostasis of the cells. Hence, autophagy has been widely established as a tumor-suppressive mechanism, and cancer cells can escape from extensive autophagic cell death resulting in the enhancement of ROS production, genomic instability, and tumor progression [54]. Defects in autophagy cause the accumulation of abnormal mitochondria that are a potential source of ROS that lead genomic instability, and cancer initiation and progression [52]. Moreover, autophagy dysfunctions cause also activation of the DNA damage response, DNA copy number variations, and genetic instability, which lead to the acquisition of genome mutations that drive tumorigenesis [53]. This situation of chronic tissue damage also provokes an inflammatory response that can, in turn, sustain tumor growth through cytokine and chemokine productions [54]. Thus, tumor promotion conferred by autophagy defects may result from both mutagenesis and the creation of an inflammatory environment. In this sense, it has been reported that mice having monoallelic deletion of the autophagy-related gene beclin1 develop spontaneous tumors. Allelic loss of beclin1 was also observed in 40–75% of breast, ovarian, and prostate cancers [3, 55]. In addition, accumulation of autophagy adaptor protein, p62/SQSTM1, as a result of the inhibition of autophagy, was reported to be responsible for the tumorigenesis by multiple mechanisms [56].

This evidence strongly suggests that autophagy is an important mechanism that suppresses tumor initiation and, when impaired, may lead to tumorigenesis. Recently, several studies have demonstrated that mutant p53 proteins exert oncogenic ability that leads to high genomic instability, reduced response to chemotherapy and generally poor clinical outcome of cancer patients [57]. Interestingly, contrarily to the wild-type counterpart, mutant p53 proteins can inhibit autophagy machinery through the stimulation of mTOR signaling pathway [58, 59] and the inhibition of AMPK [60, 61]. This oncogenic ability represents an essential turning point to sustain cancer cell proliferation and growth. On the other side, autophagy can trigger mutp53 protein degradation in a functional interplay that can regulate tumor progression and the response to antitumor therapies [62].

### Autophagy in other diseases

Neurodegenerative diseases, such as Parkinson’s disease (PD), Alzheimer’s disease (AD), Huntington’s disease (HD), and amyotrophic lateral sclerosis (ALS), are characterized by the progressive loss of neurons and/or neuronal functions. These syndromes are developed by the presence of aggregate-prone neurotoxic proteins, in the form of aggresomes and/or inclusion bodies, as well as by the presence of dysfunctional mitochondria [63]. In this regard, autophagy protects against neurodegenerative diseases acting as a quality-control system and by removing protein aggregates and damaged organelles. Therefore, it represents an essential process for the maintenance of neuronal homeostasis [64]. Recent findings have highlighted that autophagy has a crucial role also in the control of muscle mass [65]. Overstimulated autophagy is harmful to myofiber health and is involved in some inherited muscle diseases [65]. However, also defective autophagy clearance favors the aggregation of misfolded proteins and damaged organelles, and contributes to the pathogenesis of different forms of muscular dystrophies and congenital myopathies [65–67]. Interestingly, recent observations suggest that re-establishing a proper autophagic flux with pharmacological treatments might represent a promising strategy for counteracting muscle loss in muscular disorders [68]. Autophagy pathway plays a central role in the cellular quality control, metabolic adaptation and clearance of misfolded proteins and/or damaged organelles in a plethora of human diseases, and the pharmacological modulation of this process may represent a valid therapeutic challenge [77].

### Current cancer treatments based on autophagy modulation

In tumors with enhanced autophagy, as a mechanism of survival and chemoresistance, the inhibition of autophagy can suppress the ability of tumor cells to survive under cellular metabolic stress [69, 70] making them more prone to initiate cell death mechanisms. In this sense, there are a variety of examples showing that autophagy inhibitors, when used in combination with anticancer drugs, may sensitize chemoresistant cells, thus inhibiting tumor survival [47, 71]. For example, it has been reported that inhibition of autophagy function by depletion of Atg5, Atg7 or beclin1 may revert the acquired resistance against tamoxifen in HER-positive breast cancer cells [72]. The combinatorial treatment consisting of autophagy inhibitor 3-methyl-adenine (3-MA) and trastuzumab (Tmab) in HER2-positive breast cancer cells can increase the potency of chemotherapy [73]. Increased autophagy is also associated with cisplatin resistance in ovarian cancer cells, and Atg5 deletion in these cells induces apoptotic cell death [74].

**Fig. 2:**
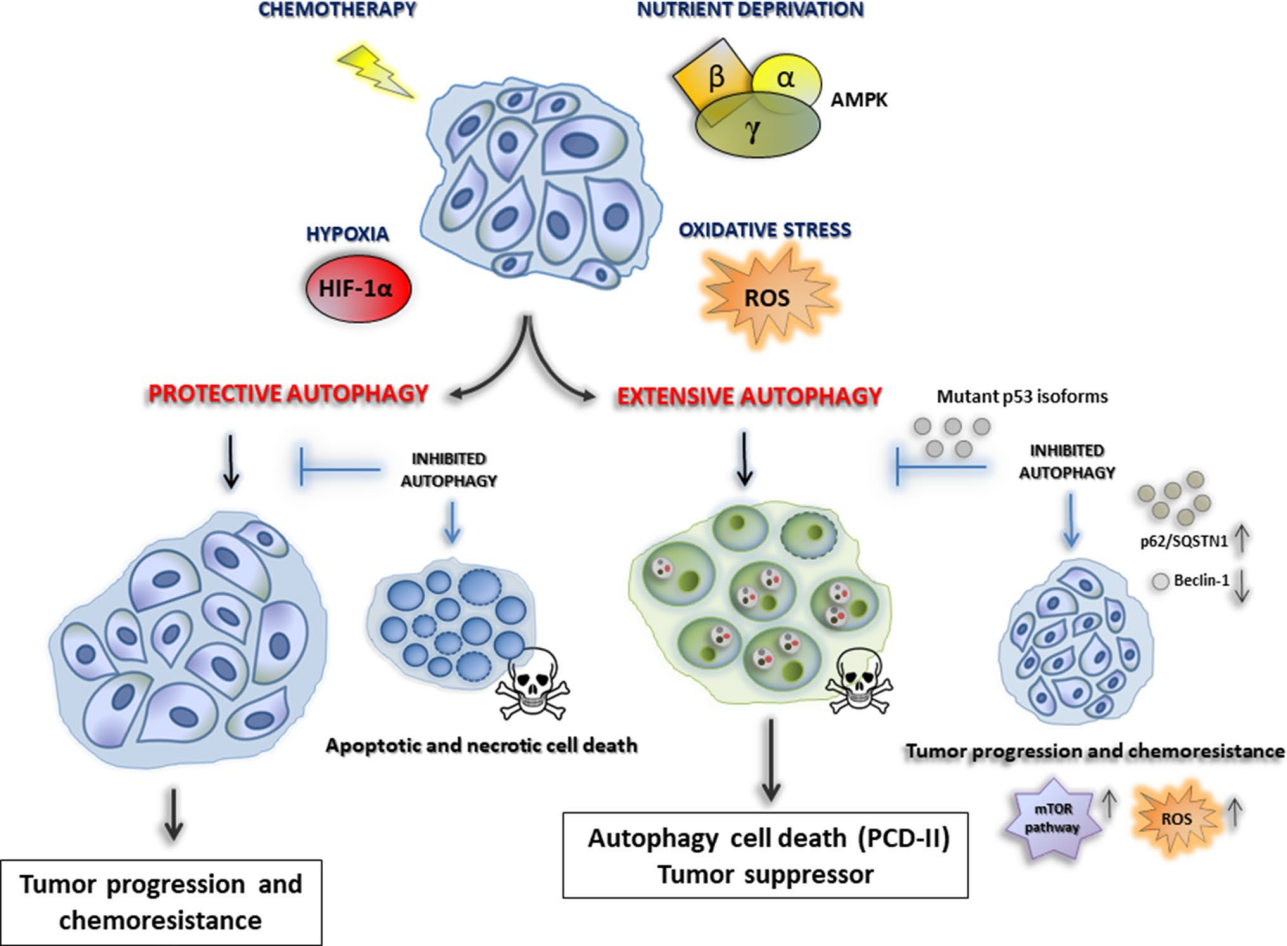
The dual role of autophagy in cancer. A variety of cellular stresses, including (1) nutrient deprivation, (2) oxidative stress, (3) hypoxia and (4) chemotherapy, can result in the induction of a protective autophagy leading tumor progression and chemoresistance. However, the same stresses can also induce and autophagy with tumor suppressor role. Indeed, cancer cells having an uncontrolled extensive autophagy can also undergo cell death-type II, likely due to excessive degradation of cellular constituents and organelles. Importantly, the inhibition of protective autophagy leads to apoptotic and necrotic cell death. In contrast, the inhibition of autophagy cell death (for instance by oncogenic mutant p53 isoforms) may lead to tumorigenesis through mTOR signaling and ROS

Among the multiple compounds that inhibit the different phases of autophagy, nowadays the only clinically approved autophagy inhibitor by the FDA is the anti-malarial chloroquine (CQ) and its derivatives, such as hydroxychloroquine (HCQ) [75]. HCQ belongs to the class of lysosomotropic agents that can inhibit lysosomal acidification and prevent the degradation of autophagosomes, thereby suppressing autophagy [76]. HCQ has been shown to have antineoplastic effects in numerous preclinical experiments when combined with other agents [77]. In renal cell carcinoma lines, HCQ enhanced the cytotoxicity of mTOR inhibitor, such as temsirolimus, promoting apoptosis and causing the downregulation of phospho-S6 through a mechanism not found in other autophagy inhibitors, such as bafilomycin A1 [78]. In breast cancer cells, the combination of HCQ and tamoxifen (TAM) was more effective at inhibiting autophagy than monotherapy in estrogen receptor-positive (ER+) breast cancer cell lines [79]. Frequently, cancer cells treated with chemotherapy drugs exhibit autophagy induction that serves as survival mechanism. However, excessive autophagy induction upon cytotoxic drug treatment or using autophagy inducers may also lead to autophagic cell death. It has been reported that glioblastoma cells resistant to apoptosis die when treated with alkylating agents such as temozolomide and tyrosine kinase inhibitors such as dasatinib, where both induce autophagy [80]. Similarly, histone deacetylase (HDAC) inhibitors have also shown autophagy-inducing potential as one of its anticancer effects [81]. Proteasome inhibitors (PI) have also been shown to stimulate autophagy. Bortezomib, a PI used in the treatment of multiple myeloma and mantle cell lymphoma, has been shown to increase the early formation of autophagosomes and LC3-II, demonstrating the inducing effects on autophagy [82]. A well-known class of autophagy inductors includes analogs of the mTOR inhibitor rapamycin, such as temsirolimus and everolimus. These compounds, used alone or in conjunction with chemotherapy drugs, show an antiproliferative effect in mantle cell lymphoma and acute lymphoblastic leukemia by overstimulating autophagy, which might cause tumor cell death [83, 84]. Everolimus has been approved for use by the FDA as an angiogenesis inhibitor in renal cell carcinoma, advanced breast cancer, and pancreatic neuroendocrine tumors [85, 86]. However, it is not clear whether the induction of autophagy by everolimus contributes to its anti-angiogenesis ability. However, in clinical trials, current targeted anticancer treatments based exclusively on mTOR inhibition have demonstrated high resistance rates [87, 88] (Table 1). Numerous ongoing phase I/II clinical trials are investigating the combination of HCQ with mTOR inhibitors in renal cell cancer, multiple myeloma and advanced solid tumors as reviewed by Duffy et al. [71]. The large number of ongoing trials demonstrates the enormous relevance that autophagy modulation can have, in combinatory treatments, to overcome the resistance to existing cancer therapies (Fig. 2).

**Table 1:** Some examples of current cancer treatments based on autophagy modulation

Entry	Drugs	Target	Type of cancer	Status of the study	Effect on autophagy	Biological effect	References
1	Chloroquine	Lysosomes	Breast cancer	Approved by FDA	Inhibitory	Inhibition of a protective autophagy	[75]
2	Hydroxychloroquine	Lysosomes	Esophageal, hepatocellular carcinoma, lung, pancreatic cancer	Approved by FDA	Inhibitory	Inhibition of a protective autophagy	[75]
3	Hydroxychloroquine + erlotinib	Lysosomes, EGFR	Advanced non-small cell lung cancer	Phase I clinical trial	Inhibitory	Safe and well tolerated	[77]
4	Hydroxychloroquine + temsirolimus	Lysosomes, mTOR pathway	Metastatic renal cancer	Cancer cell lines	Inhibitory	Apoptosis, mitochondrial damage, mTOR downregulation	[78]
5	Hydroxychloroquine + tamoxifen	Lysosomes, estrogen receptor-α (ERα)	Breast cancer	Cancer cell lines, in vivo models, phase I clinical trial	Inhibitory	Reduced drug resistance and potentiation of antiestrogenic therapy in vitro and in vivo	[79]
6	Temozolomide, dasatinib	Alkylating agents, tyrosine kinase inhibitors	Glioblastoma	Cancer cell lines, phase I clinical trial	Induction	Increased therapeutic efficacy of temozolomide, apoptosis of resistance cells	[80]
7	Histone deacetylase inhibitors	Histone deacetylase	Hepatocellular carcinoma	Cancer cell lines	Induction	Autophagy cell death	[81]
8	Bortezomib	Proteasome	Prostate cancer	Cancer cell lines	Induction	Autophagy protective	[82]
9	Temsirolimus + vorinostat	mTOR pathway, histone deacetylase	Lymphoma	Cancer cell lines	Induction	Synergistic antiproliferative activity, apoptosis, autophagy cell death	[83]
10	Everolimus + vincristin	mTOR pathway, tubulin	Acute lymphoblastic leukemia	In vivo models	Induction	Synergistic tumor growth reduction, cell death, and survival of engrafted mouses	[84]
11	Rapamycin, everolimus	mTOR pathway	Cancer metastasis	In vivo models	Induction	Metastatic tumor growth, angiogenesis, reduced cancer recurrence	[85]
12	Everolimus	mTOR pathway	Metastatic renal cell carcinoma	Phase III clinical trial	Induction	Prolongation of tumor-free survival	[86]
13	Hydroxychloroquine + temsirolimus	mTOR pathway	Melanoma	Three-dimensional spheroid cultures, in vivo models	Induction	Tumor growth suppression, cell death, apoptosis	[87]
14	Everolimus	mTOR pathway	Lymphoma	Cancer cell lines	Induction	Protective autophagy, drug resistance	[88]

## Nanomaterials as therapeutic tools

Recent progress in nanotechnology offers plenty of tools to fight cancer with innovative, personalized and efficient therapeutic agents by overcoming barriers or drawbacks usually encountered with traditional drugs [89]. The recent advances in the field of chemistry and material science have produced nanomaterials which are expected to improve the treatment of many diseases otherwise resistant to the traditional therapeutic approaches. Nanomaterials can act as cytotoxics and/or enhance the efficacy of standard chemotherapies. Moreover, they represent novel drug delivery systems thereby decreasing the side effects of conventional drugs. These nanomaterials exhibit unique physical, chemical, mechanical and optical properties that make them suitable as novel and powerful therapeutic tools. Depending on their morphology, size and chemical properties, nanomaterials are broadly divided into various categories, including liposomes, carbon-based materials, polymers, metals, metals oxide, and ceramics. Most of these nanomaterials are used as nanocarriers to deliver therapeutic molecules, such as drugs, proteins or nucleic acids [90–99].

**Fig. 3:**
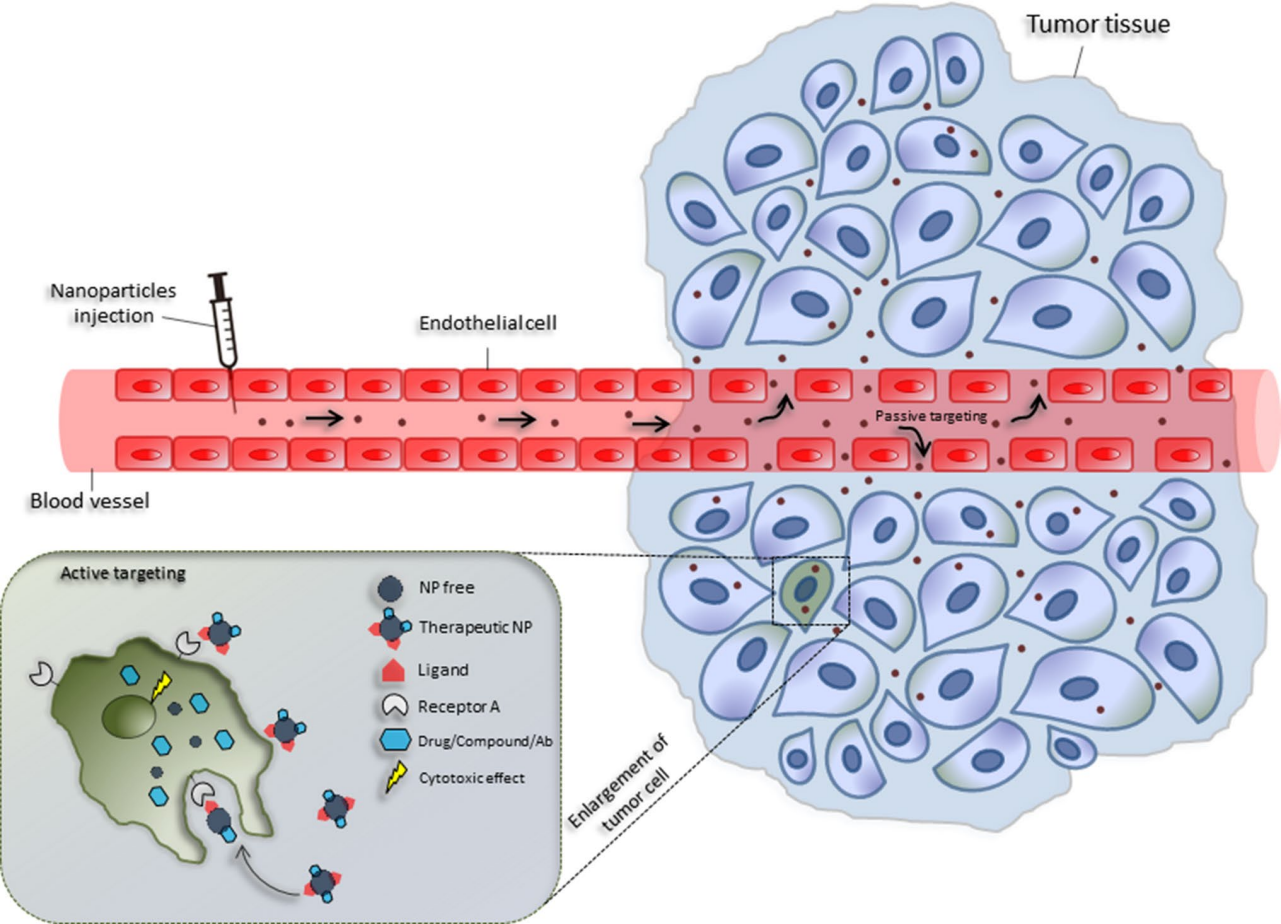
Passive and active targeting of nanoparticles in cancer treatment. Passive tumor targeting is achieved by extravasation of nanoparticles through increased permeability of the tumor vasculature (EPR effect). Active tumor targeting (left inset) can be achieved by functionalization of nanoparticles with targeting ligands that promote cell-specific recognition and binding. Once internalized, the nanoparticles can express their cytotoxic potential by releasing the drug and/or another compound

One of the major challenges of nanomedicine consists of developing drug delivery systems to transfer drugs, proteins, enzymes or antibody into specific target sites without affecting healthy tissues. The primary physical, chemical, and biological advantages in the use of nanocarriers include the nanoscale sizes, high surface-to-volume ratios, favorable drug release profiles and targeting modifications [6]. Interestingly, nanocarriers can passively accumulate in the leaky vasculature, typical of tumor tissues in a manner known as the enhanced permeability and retention effect (EPR) [100]. Nanocarriers may also be conjugated with specific ligands, to utilize active targeting mechanisms [101]. This allows them to reach specific tissues and release drugs in a stable and controlled manner. Therefore, through active targeting, the use of nanomaterials in drug delivery can modify the pharmacokinetic and pharmacodynamic profiles of drugs, thereby enhancing their therapeutic index [100, 102]. Microenvironmental stimuli can trigger the release of drugs by evoking a change in the nanocarriers, to ensure specific toxicity to the target tissue, while does not affect the healthy tissue [10]. Changes in pH, redox, ionic strength, and stress in target tissues are examples of internal stimuli [7, 13]. External stimuli, including temperature, light, ultrasound, magnetic force, and electric fields, also can trigger the release of drugs [7] (Fig. 3).

Among the different nanostructures, metallic nanoparticles are particularly relevant due to their inherent reactivity, which can be used for therapeutic purposes, such as hyperthermia. Hyperthermia-based approaches [103] consist of exposing the body to high temperatures to kill cancer cells or sensitizing them to the effects of radiation and certain anticancer drugs. The use of metallic nanoparticles allows for the application of a variety of techniques such as laser, ionizing radiation and microwaves, to induce heat at the nanoparticles area [103]. Magnetic hyperthermia allows to remotely induce local heat using the magnetic energy losses of magnetic nanoparticles under an alternate magnetic field (AMF), thus drastically reducing the harmful side effects at the surrounding healthy tissues [104]. Importantly, a number of studies describe magnetic hyperthermia as a very attractive adjuvant strategy to radiation and chemotherapy in cancer treatment [105]. Alternatively, by exposing metallic nanoparticles to laser radiation near their plasmon-resonant absorption band, it is possible to produce local heating of nanoparticle-labeled cells without harming surrounding healthy tissues. Such some promising approaches, which induce the photothermal effect in vitro and in vivo, have been developed over the last years. They include plasmonic photothermal therapy (PPTT) [106], and red and near-infrared (NIR) laser light irradiations [107]. Interestingly, massive cancer cell death, reduction of tumoral volume and general improvements in survival have been demonstrated when the gold nanoparticles are actively targeted to tumors in mice over passively targeted nanoparticles [108–111].

## Targeting autophagy with metal-based nanoparticles as therapy in cancer diseases

**Fig. 4:**
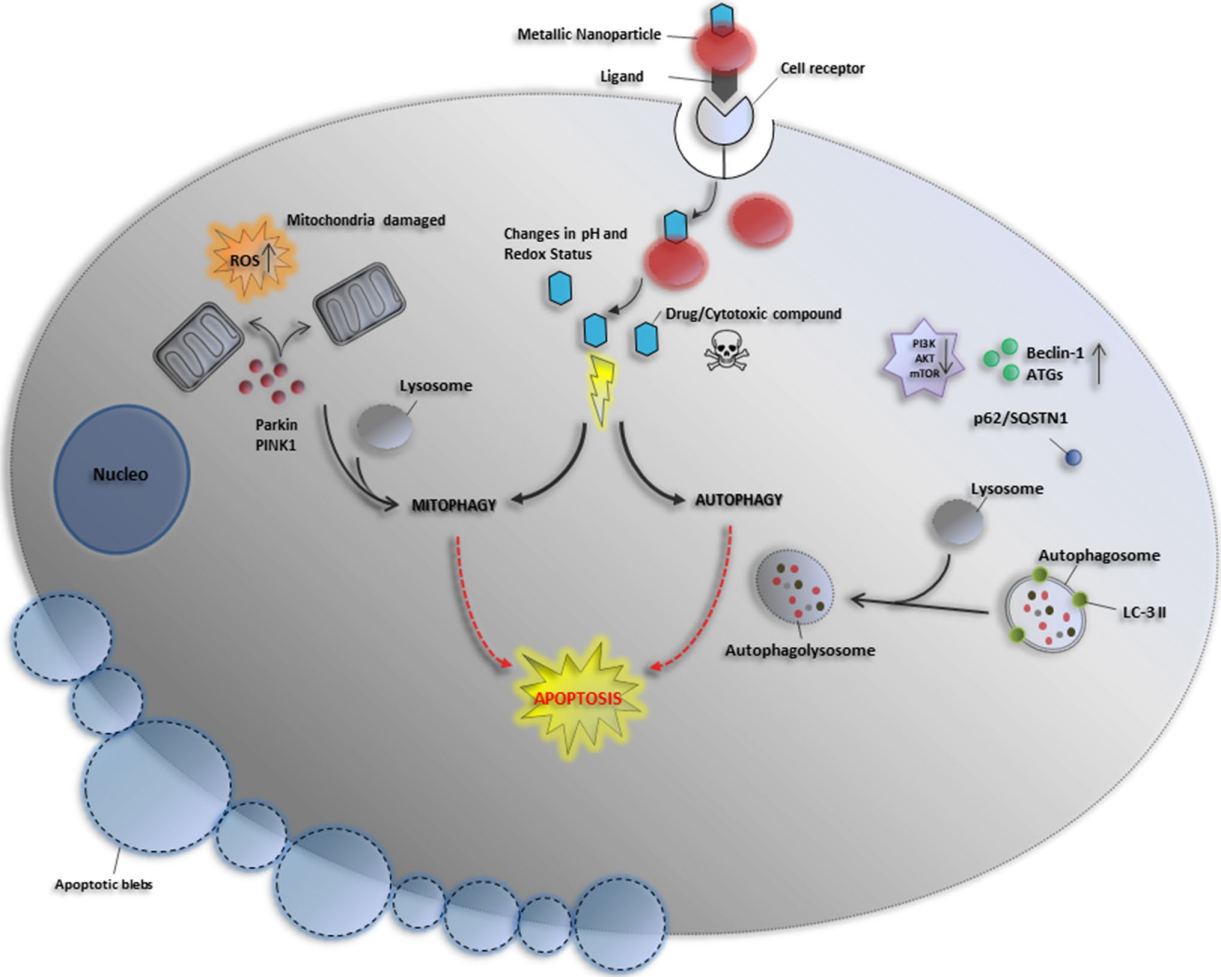
Cytosolic delivery of drug-loaded metallic nanoparticles via receptor-mediated endocytosis and its effect on autophagy and mitophagy modulation. The metallic nanoparticles, once internalization via receptor-mediated endocytosis, release the drugs or other compounds loaded, thus exerting a cytotoxic effect against cancer cells. The release occurs as a consequence of some cellular environmental stimuli, such as changes in pH and redox status by evoking changes in the nanocarrier structure. The toxic effect is exerted in various manners by inducing mitochondrial damage, and autophagy and mitophagy processes that culminate with apoptotic and autophagic cell death

In this section, we summarize the most recent studies describing the ability of metallic nanoparticles to overstimulate autophagy and mitophagy in cancer cells selectively. This occurs through the dysregulation of some cellular signaling pathways without significantly affecting the level of autophagy in noncancerous cells. The effect of several nanomaterials on autophagy/mitophagy modulation represents an exciting therapeutic approach against different human tumors (Fig. 4).

### Silver-based nanoparticles

Different studies showed the enormous therapeutic potential of silver nanoparticles (Ag-NPs) against a plethora of cancer cells. It has been reported that these nanomaterials can modulate autophagy acting as cytotoxic agents itself, in combination with other treatments, as well as nanocarriers to deliver therapeutic molecules [112–120]. For example, it has been shown that Ag-NPs, embedded into a specific exopolysaccharide (EPS), exert a cytotoxic effect against a panel of cancer cell lines. This occurs through the promotion of ROS which, in turn, induced cell death through apoptosis and autophagy stimulations. These observations were further confirmed in SKBR3 cells after Ag-NPs–EPS exposure in which the induction of autophagic markers was detected by fluorescence microscopy and western blot indicating a prominent mechanism of autophagic cell death [112].

In another study, Ag-NPs have been observed to have a higher cytotoxic effect on PANC1 cancer cells compared to the non-tumor cell of the same tissue. In particular, Ag-NPs decreased the viability of PANC-1 cells and stimulated apoptotic and autophagic cell death more significantly than non-tumor cells. Moreover, the authors observed that the protein level of autophagy marker LC3-II increased substantially in PANC-1 cells treated with Ag-NPs, thus indicating that the apoptotic and necroptotic cell death is occurring with autophagy in adenocarcinoma pancreatic cancer cells [113].

In a related report, Cisplatin and a reduced graphene oxide–silver nanoparticle nanocomposite (rGO–Ag-NPs) were assessed in HeLa cancer cells. Interestingly, the combination of Cis and rGO–Ag-NPs resulted in more pronounced effects on the expression of autophagy genes and in the accumulation of autophagosomes and autophagolysosomes, which were associated with the generation of ROS and cell death. These findings demonstrate that rGO–Ag-NPs can potentiate Cis-induced cytotoxicity, apoptosis, and autophagy in HeLa cells, and hence rGO–Ag-NPs could be potentially applied to cervical cancer treatment as a powerful synergistic agent with Cis or any other chemotherapeutic agents [114]. Notably, also the combination of Salymicin (Sal) and Ag-NPs showed a substantial synergistic effect on cytotoxicity and in the accumulation of autophagolysosomes in A2780 ovarian cancer cells. The induction of massive autophagy, in turn, led to mitochondrial dysfunction and cell death, thus representing a relevant therapeutic strategy for the treatment of ovarian cancer [115].

Moreover, it has been found that the combination of Ag-NPs and radiotherapy significantly enhanced cytotoxic effects in U251 glioblastoma cells and orthotopic mouse brain tumor model. In addition, LC3-II protein level, acridine orange (AO) and monodansylcadaverine (MDC) staining revealed that autophagy was strongly upregulated following the treatment of Ag-NPs with ionizing radiation, suggesting that modulation of the autophagy process may improve glioblastoma therapeutic outcome [116]. Transcription factor EB (TFEB) is a master regulator of lysosomal biogenesis, and it has been reported to regulate autophagy by upregulating a cluster of autophagic genes, including MAPLC3B, SQSTM1, UVRAG, WIPI, VPS11, VPS18, and ATG9B [121, 122]. Recently, Ag-NPs have been shown to reduce the expression of TFEB in A459 lung cancer cells, thus affecting lysosome function and autophagic flux, and leading cellular damage [123].

### Gold-based nanoparticles

Gold nanoparticles (Au-NPs) have been extensively explored in biomedical research as drug delivery scaffolds, because of their low toxicity and immunogenicity [124, 125], good biocompatibility and excellent stability [126]. Remarkably, the surface of Au-NPs can be easily modified with multiple agents including chemotherapeutics, oligonucleotides, and proteins, making them excellent delivery vehicles.

It has been reported that pH-sensitive polymeric nanoparticles with gold(I) induce cell death in MCF7 breast cancer cell death through regulation of oxidative stress and autophagy [127]. A recent study describes the development of SMI#9-tethered Au-NPs using a chemical strategy that allows the intracellular release of SMI#9, a small inhibitor of Rad6, a central player in DNA damage tolerance, post-replication DNA repair mechanism and mitochondrial stability [128, 129]. The authors of this study observed an increase in autophagy and apoptotic markers in SUM1315 triple-negative breast cancer (TNBC) cells after treatment with SMI#9-AuNPs, suggesting an essential role for Rad6 in assuring the survival of cancer cells [130].

In a related study, gold nanoparticles were modified with the snake venom protein toxin NKCT1 (Au-NPs–NKCT1) and tested in human leukemic U937 and K562 cell lines [131]. The authors reported that Au-GNPs–NKCT1 treatment exerts its cytotoxic potential by inducing a caspase 3-mediated apoptosis and an autophagic cell death response due to the dysregulation of AKT/mTOR signaling pathways. Therefore, the conjugation of Au-NPs with NKCT1 represents a promising strategy to develop therapies from natural resources such as snake venoms [131].

It has been reported that Au-NPs, in combination with tumor necrosis factor (TNF)-related apoptosis-inducing ligand (TRAIL), were able to promote a relevant Drp1-mediated mitochondrial damage leading apoptosis, autophagy and mitophagy activation [132]. In support of this, the authors found that mitophagy markers PINK1 and Parkin were recruited into mitochondrial fractions and autophagy signature was detected in Calu-1 cells after combined treatment [132]. Hence, autophagy and mitophagy activations in response to TRAIL combined with Au-NPs may represent a strategy to overcome TRAIL resistance that occurs in many tumors.

The epidermal growth factor receptor (EGFR) is overexpressed in 70–80% of TNBC and has been emerging as a promising target for TNBC treatment [133]. In a recent report, the role of autophagy was assessed in the cytotoxicity induced by anti-EGFR antibody-conjugated gold nanoparticle (anti-EGFR–Au-NPs)-combined near infrared-photothermal therapy (NIR-PTT) in MDA-MB-231 cancer cells [134]. Interestingly, the cell death induced by anti-EGFR–Au-NPs-combined NIR-PTT was rescued by treatment with 3-MA. Anti-EGFR–Au-NPs-combined NIR-PTT strongly induced autophagy as evidenced by autophagic vesicles and a significant increase in several autophagy-related markers, accompanying the inhibition of AKT/mTOR signaling pathway. In addition, in mouse xenograft tumors, anti-EGFR–Au-NPs-combined NIR-PTT also increased LC3 and beclin-1 levels. These findings demonstrate that autophagy elicited by anti-EGFR–GNs-combined NIR-PTT is an alternative cell death mechanism, resulting in most effective cancer therapy for EGFR-targeted TNBC [134].

Tmab is a humanized monoclonal antibody routinely used for patients with HER2-positive breast and gastric cancers that improves survival [135]. In a recent study, Au-NPs modified with Tmab were evaluated in NCI-N87 and MKN7 HER2-positive gastric cancer. Interestingly, the authors report that T-Au-NPs possessed specific HER2-based tumor selectivity and exerted a potent cytotoxic effect through the induction of autophagy mechanism that differs from those of the non-conjugated Tmab [136].

Quercetin (3,3′,4′,5,7-pentahydroxy-flavone) is a flavonoid found in a wide variety of plants and constituent in human diet [137]. Quercetin exhibits beneficial effects on human health and possesses selective antiproliferative and antitumor effects via apoptotic mechanisms on different human cancer cell lines [138]. It has been reported that gold–quercetin nanoparticles, stabilized by PLGA, induce autophagy cell death and apoptosis through dysregulation of signaling pathways in human liver, cervical and neuroglioma cancer cells [139–141].

Interestingly, it has been observed that monolayers of chiral molecules anchored on the surfaces of Au-NPs (d-PAV–Au-NPs; l-PAV–Au-NPs) induced chirality-selective autophagy selectively in MDA-MB-231 cancer cells. Furthermore, the intratumoral injection of d-PAV–Au-NPs suppresses the tumor growth without side effects in vivo [142]. This specific effect was likely attributed to the chirality-variant ROS generation, cellular uptake, and their continuous autophagy stimulus.

Layered nanoparticles made with an iron core and a gold shell, Fe@Au, combine the strong magnetic susceptibility of pure iron and the passivating properties of the gold coating. In practice, the gold shell only delays the oxidation, rather than stopping it entirely [143]. Fe@Au-NPs have been reported to exert toxicity in oral and colorectal cancer cells through mitochondria-mediated autophagy and, therefore, have been proposed as a potential anticancer agent [144, 145].

### Metal oxide-based nanoparticles

Zinc oxide nanoparticles (ZnO-NPs) are routinely used in industrial products, and more recently, they have been employed in biomedical and cancer applications due to the attractive chemical properties of these nanomaterials [146]. In a recent study, it has been reported that ZnO-NPs can induce significant cytotoxicity, apoptosis, and autophagy in SKOV3 ovarian cancer cells via induction of intracellular ROS and oxidative stress [147]. ZnO-NPs have also been shown to induce toxicity in CAL27 oral cancer cell lines by activating PINK1/Parkin-mediated mitophagy [148].

It has been reported that conjugation of ZnO-NPs with meso-tetra (4-carboxyphenyl) porphyrin (MTCP) could increase their cytotoxic effects through autophagy induction in MCF-7 and MDA-MB-468 breast cancer cells [149]. These reports strongly suggest a possible application of ZnO-NPs as anticancer agents.

Iron oxide nanoparticles (IO-NPs) are widely used in biomedicine for their multi-functional properties of super-paramagnetism and biocompatibility as well as in cancer treatment due to their drug delivery and multi-imaging functions [150]. However, some issues concerning their therapeutic efficiency and biological safety limited their development and clinical translation.

Interestingly, IO-NPs have been reported to induce autophagy process through multiple mechanisms including lysosome impairment, mitochondrial damage, and ER stress [151].

IO/Au-NPs conjugated to anti-EGFR suppress lung tumor growth both in vitro and in vivo, by abrogating G2/M cell-cycle arrest and inducing DNA damage, autophagy and apoptosis [152].

In an excellent study, researchers have developed chitosan chloride (HTCC)/alginate-encapsulated Fe_3_O_4_ NPs (HTCC–MNPs) and applied them to multi-drug resistance (MDR) gastric cancer models. Interestingly, they reported that the novel HTCC–MNPs were more cytotoxic in both SGC7901 human gastric cancer cell line and MDR variant cell line (SGC7901/ADR) than to normal gastric epithelial cell line (GES). In addition, the co-localization of LC3 with lysosomal marker LAMP2 and an increased LC3-II/LC3-I ratio revealed the induction of autophagy by HTCC–MNPs. Therefore, these data indicated that autophagy was responsible for the cytotoxicity induced by HTCC–MNPs, highlighting that its modulation may have a role in treating MDR gastric cancer [153]. Moreover, IO-NPs have been shown to selectively induce significant autophagy–lysosome accumulation and cell death through dysregulation of Akt/AMPK/mTOR pathway and in a dispersity-dependent manner, in lung and cervix cancer cells but not in normal cells [154, 155].

Another study has reported that PEGylated IO-NPs caused severe cytotoxicity in SKOV3 human ovarian cancer cells through multiple mechanisms, such as ROS production and apoptosis induction. Notably, the authors observed changes in autophagosome formation when SKOV3 cells were exposed to PEGylated-IO-NPs by TEM imaging and by detecting the level of autophagy marker LC3-II. The authors concluded that autophagy induction could be a protective role against cytotoxicity IO-NPs-induced [156].

In another study, it has been shown that IO-NPs photothermal effect could lead to autophagy induction in both MCF-7 cancer cells and MCF-7 xenograft model, in a laser dose-dependent manner, and the inhibition of autophagy would enhance the photothermal cell killing by increasing cell apoptosis. Therefore, this work may provide a potential combination therapeutic approach of autophagy modulators and photothermal agents [157].

Cuprous (Cu-NPs) and copper oxide nanoparticles (CO-NPs), are other nanomaterials with biomedical application, which showed potential pharmacological effects on tumor therapy by inducing apoptosis, inhibiting metastasis and stimulating autophagic cell death in leukemia, melanoma, and lung and breast cancers [158–161].

In a study by Xia et al., Cu-NPs dramatically affect autophagy pathway in human cervical cancer cell lines, thus leading to inhibition of cell growth and apoptosis. In particular, the authors reported that Cu-NPs could decrease the phosphorylation of AKT and mTOR, strongly suggesting that Cu-NPs could induce autophagy through AKT/mTOR pathway. Moreover, they observed the increase of the autophagosome formation in a time- and concentration-dependent manner. Their work provides preliminary evidence of the therapeutic potential of CO-NPs in the treatment of cervical cancer [162]. Also, CO-NPs have been reported to induce autophagy in MCF7 human breast cancer cell line, in a time- and dose-dependent manner. The authors of the study hypothesize that autophagy induced by CO-NPs may serve as a cellular defense against their intrinsic toxicity, and inhibition of autophagy could be essential to induce apoptosis in breast cancer cells [163].

### Silica-based nanoparticles

Many studies have demonstrated that amorphous silica nanoparticles (Si-NPs) possess unique properties such as biocompatibility, tunable pore size, high surface area, and ease of modification. For this reason, Si-NPs have been widely used in gene transfection, drug delivery, biosensing and bioimaging [164–166]. It has been observed that these nanostructures promote osteoblast differentiation through autophagy stimulation [167]. Si-NPs have also been shown to stimulate ROS generation, oxidative stress and ER stress that lead to autophagy activation via unfolded protein response (UPR) pathways in hepatocytes [168].

Several other studies reported that through autophagy modulation, Si-NPs exert cytotoxic effect in cancer cells thus highlighting their potential therapeutic effect. It has been reported that Si-NPs can lead to apoptosis, mitophagy, autophagy, and consequently ROS accumulation in glioblastoma LBC3 cells representing a potential therapeutic agent for glioblastoma multiforme therapy [169].

Intriguingly, it has been shown that accumulation of Si-NPs in human cervix carcinoma cells may lead to lysosomal dysfunctions and autophagy defects, resulting in a reduced metabolic activity of cancer cells [170]. Recently, genistein–PEGylated silica hybrid nanomaterials (Gen–PEG–SiHNM) have been developed, which possess antiproliferative effects by activating apoptosis and autophagy cell death in HT29 human colon cancer cells [171]. This study suggests that Gen–PEG–SiHNM may be potentially used as an alternative treatment for colorectal cancer in the near future.

Si-NPs have also been observed to induce ROS and autophagy dysfunction in HCT-116 colon cancer cells, L-02 and HepG2 hepatoma cells, providing novel evidence for the study of toxic effect and safety evaluation of Si-NPs [117, 172–174]. Endoplasmic reticulum-involved autophagic process (ER autophagy) is a selective form of autophagy in which these organelles can be captured by autophagy process if they are damaged [175, 176]. Recently, it has been observed that Si-NPs induce endoplasmic reticulum (ER) autophagy in HCT-116 human colon cancer cells without exhibiting a strong cytotoxic effect. The autophagy induced by these nanomaterials was detected by the increase of LC3-II and was associated with the treated time but not by the concentration used. These new findings of Si-NPs-induced ER autophagy could open an effective way for securely designing silica-based nanoparticles and could potentially represent a valid therapeutic tool in autophagy-linked diseases [174] (Table 2).

**Table 2:** NP-Mediated Autophagy in cancer cells

Entry	NP	Compound carried/combination drug	Target Cells	Cancer tissue type	Autophagy markers	Autophagy mechanism	Biological effect	References
1	Silver	EPS (exopolysaccharide)	SKBR3	Breast cancer	LC-3 ↑	Not reported	Autophagy, ROS, cell death	[112]
2	Silver	None	PANC1	Pancreatic cancer	LC-3 ↑	Not reported	Autophagy, ROS, apoptosis, necrosis, cell death	[113]
3	Silver	Reduced graphene-oxide	HeLa	Cervical cancer	LC-3 ↑, ATGs ↑	Not reported	Autophagy, ROS, cell death	[114]
4	Silver	Salymicin	A2780	Ovarian cancer	ATGs ↑	Not reported	Autophagy, mitochondrial dysfunction, cell death	[115]
5	Silver	Radiotherapy	U251	Glioblastoma	LC-3 ↑, MDC ↑	Not reported	Autophagy, ROS, apoptosis, cell death	[116]
6	Silver	None	HepG2	Liver cancer	LC-3 ↑	Not reported	Autophagy flux, lysosomal activity, apoptosis	[118]
7	Silver	None	A549	Lung cancer	LC-3 ↑, ATG-5 ↑; p62 ↑	Not reported	Autophagy, apoptosis, mitochondrial damage, cell death	[119]
8	Silver	Fungal-derived protein-capped	Huh7, HOS CRL-1543	Liver cancer, osteosarcoma	LC-3 ↑	Not reported	Autophagy, ROS, apoptosis, cell death	[120]
9	Silver	None	A549	Lung cancer	LC-3 ↑; p62 ↑	Transcription Factor EB ↓	Autophagy flux inhibition, lysosome impairment	[123]
10	Gold	pH sensitive mPEG-poly(amino ester)s graft copolymers	MCF7	Breast cancer	LC-3 ↑	Not reported	Autophagic cell death, lysosome impairment	[127]
11	Gold	SMI#9	SUM1315	Breast cancer (TNBC)	LC-3 ↑; p62↓	Not reported	Autophagy, apoptosis, cell death	[130]
12	Gold	Venom protein toxin (NKCT1)	U937, K562	Leukemia	LC-3 ↑, Beclin-1 ↑, ATG-3 ↑, ATG-12↑	PI3K/Akt/mTOR ↓	Autophagy, apoptosis, cell death	[131]
13	Gold	TRIAL	Calu-1	Non-small lung cancer (NSCLC)	LC-3 ↑	not reported	Autophagy, mitochondrial dysfunction, mitophagy, ROS, apoptosis, cell death	[132]
14	Gold	Anti-EGFR + NIR-PTT	MD-MB-231	Breast cancer (TNBC)	LC-3 ↑, Beclin-1 ↑, ATG-5 ↑, p62 ↓	Akt/mTOR ↓	Autophagy, cell death	[134]
15	Gold	Tmab	NCI-N87, MKN7	Breast cancer, Gastric cancer	not reported	Akt/mTOR ↓	Autophagy, cell death	[136]
16	Gold	Quercetin into poly (dl-lactide-*co*-glycolide)	MHCC97H, Hep3B, HCCLM3, Bel7402	Liver cancer	not reported	Akt↓, ERK1/2↓	Apoptosis, cell death	[139]
17	Gold	Quercetin into poly (dl-lactide-*co*-glycolide)	U87	Glioma cancer	LC-3 ↑, Beclin-1 ↑, ATG-1 ↑, p62 ↓	Akt/mTOR ↓, ERK1/2↓	Autophagy, apoptosis, cell death	[140]
18	Gold	Quercetin into poly (dl-lactide-*co*-glycolide)	Caski, HeLa, Siha	Cervical cancer	LC-3 ↑	mTOR ↓	Autophagy, apoptosis, cell death	[141]
19	Gold	Poly (acryloyl-l, d and racemic valine)	MDA-MB-231	Breast cancer	LC-3 ↑	Not reported	Autophagy, ROS, cell death	[142]
20	Gold/iron	Iron core, gold shell	OECM1	Oral cancer	LC-3 ↑	Not reported	Autophagy, apoptosis, ROS, mitochondrial damage, cell death	[144]
21	Gold/iron	Iron core, gold shell	Caco-2, HT-29, SW480	Colorectal cancer	LC-3 ↑	Not reported	Autophagy, apoptosis, ROS, mitochondrial damage, cell death	[145]
22	Zinc oxide	None	SKOV3	Ovarian cancer	LC-3 ↑	Not reported	Autophagy, apoptosis, ROS, cell death	[147]
23	Zinc oxide	None	CAL27	Oral cancer	LC-3 ↑, p62 ↓	PINK1/Parkin↑	Autophagy, apoptosis, ROS, mitophagy, cell death	[148]
24	Zinc oxide	Meso-tetra (4-carboxyphenyl) porphyrin	MCF-7, MDA-MB-468	Breast cancer	LC-3 ↑, Beclin-1 ↑, ATG-3↑, DRAM-1↑	Not reported	Autophagy, apoptosis, cell death	[149]
25	Iron oxide	None	MCF-7	Breast cancer	LC-3 ↑, ULK-1 ↑, p62 ↓	mTOR ↓	Autophagy, ROS, mitochondrial damage, ER stress, apoptosis, cell death	[151]
26	Iron oxide/gold	Anti-EGFR antibody	HCC827	Lung cancer	LC-3 ↑	Not reported	Autophagy, DNA damage, apoptosis, tumor growth suppression	[152]
27	Iron oxide	Chitosan chloride (HTCC)/alginate	SGC7901, SGC7901/ADR	Gastric cancer	LC-3 ↑, LAMP1↑	Not reported	Autophagy, apoptosis, ROS, mitochondrial dysfunction, cell death	[153]
28	Iron oxide	None	A459	Lung cancer	LC-3 ↑, ATG-5↑, ATG-12↑	mTOR ↓, p70S6K, AMPK ↑	Autophagy, ROS, mitochondrial damage, apoptosis, necrosis, cell death	[154]
29	Iron oxide	None	HeLa	Cervical cancer	LC-3 ↑, p62 ↓	mTOR ↓	Autophagy	[155]
30	Iron oxide	PEG	SKOV3	Ovarian cancer	LC-3 ↑	Not reported	Protective autophagy, ROS, apoptosis, cell death	[156]
31	Iron oxide	Photothermal treatment	MCF-7	Breast cancer	LC-3 ↑, p62 ↓	Not reported	Protective autophagy	[157]
32	Cuprous oxide	None	SiHa, Caski, MS751, HeLa	Cervical cancer	LC-3 ↑	Akt/mTOR ↓	Autophagy, apoptosis, mitochondrial damage, cell death	[162]
33	Copper oxide	None	MCF7	Breast cancer	LC-3 ↑, beclin-1 ↑, ATG-5 ↑	Not reported	Protective autophagy	[163]
34	Silica	None	LBC3	Glioblastoma	LC-3 ↑, ATG-5↑	Not reported	Apoptosis, autophagy, mitophagy, ROS	[169]
35	Silica	None	HeLa	Cervical cancer	LC-3 ↑; p62 ↑	Not reported	Autophagy and lysosomal dysfunction	[170]
36	Silica	Genistein-PEGylated	HT-29	Colorectal cancer	autophagosome accumulation	Not reported	Autophagy, apoptosis, cell death	[171]
37	Silica	None	L-02, HepG2	Liver cancer	LC-3 ↑, p62↑	Not reported	Autophagy, ROS, cell death	[172]
38	Silica	None	HepG2	Liver cancer	LC-3 ↑, p62↑	Not reported	Autophagy, ROS, cell death	[173]
39	Silica	None	HCT-116	HCT-116	LC-3 ↑; p62 ↓	Not reported	Protective autophagy, ER-autophagy	[174]
40	Silver	None	B-16 mouse cell	Melanoma	LC-3 ↑; p62↓	PtdIns3K-III ↑	Protective autophagy, cell survival	[182]
41	Silver	Radiation treatment	U251	Glioma	LC-3 ↑	ROS dependent	ROS, mitochondrial damage, protective autophagy	[185]
41	Ferroferric oxide	None	K562, OCI-AML2; OPM2, RPMI-8226	Leukaemia, multiple myeloma	LC-3 ↑; p62↓	Beclin 1/VPS34/Atg14 complex ↑	Protective autophagy, drug resistance	[186]
42	Silica	None	A459	Lung cancer	LC-3 ↑	not reported	Protective autophagy, antiapoptotic genes	[187]
43	Silver	None	HeLa	Cervical cancer	MAPLC3B ↑ SQSTM1/p62 ↑; LAMP1 ↑, CTSB ↑, CTSD↑, ATP6V1H↑	mTOR ↓, transcription factor EB nuclear translocation	Protective autophagy	[191]
44	Titanium dioxide	None	HeLa	Cervical cancer	LC-3 ↑	transcription factor EB ↑	Autophagy	[206]

## Other examples of autophagy modulation using nanoparticles

The use of nanoparticles to modulate autophagy is not just limited to cancer, and a variety of maladies can be treated by this approach, such as muscular or neurodegenerative diseases. Neurodegenerative diseases are a complex set of severe disorders characterized by the progressive loss of neurons leading to severe physical and cognitive inabilities in affected patients. Impairment of autophagy machinery has been reported to be linked with the development of these diseases, and several studies have recently reported that autophagy modulation by nanoparticles may represent a valid therapeutic opportunity.

Cerium oxide nanoparticles (CeO_2_-NPs), due to their antioxidant proprieties, have been exploited for biomedical application [177, 178]. In addition to protect cells from oxidative stress, these nanomaterials were reported to activate autophagy and promote clearance of autophagic cargo, thus exerting a neuroprotective function. Interestingly, different functionalized CeO_2_-NPs have been shown to enhance autophagic clearance of proteolipid aggregates in fibroblasts derived from a patient with late infantile neuronal ceroid lipofuscinosis (LINCL) that accumulates the autophagic substrate ceroid lipopigment as a result of the inefficient function of the lysosome autophagy system. At the mechanistic level, autophagy induction by CeO_2_-NPs was due to the activation of TFEB that controls the expression of genes involved in lysosomal function and autophagy [179].

Europium hydroxide nanoparticles [(EuIII(OH)_3_)-NPs] has been shown to be able to reduce mutant huntingtin protein aggregation via autophagy induction, which is responsible for many neurodegenerative diseases. The induction of autophagy flux by [(EuIII(OH)3)-NPs] has been observed in different cell lines such as Neuro 2a, PC12 and HeLa cells, and it was due to the expression of characteristic autophagy marker LC3-II and degradation of selective autophagy substrate/cargo receptor p62/SQSTM1 [180] (Table 3).

**Table 3:** NP-Mediated Autophagy in other diseases and non-cancer cells

Entry	NP	Compound carried/combination drug	Target cells	Tissue type	Autophagy markers	Autophagy mechanism	Biological effect	References
1	Silver	polyvinylpyrrolidone (PVP)	Murine pro-B cells Ba/F3	Blood	LC-3 ↑; p62 ↓	ROS dependent, PI3K–Akt–mTOR ↓	Autophagy, DNA damage, apoptosis, cell death	[117]
2	Cerium oxide	*N*-Acetylglucosamine (GlcNAc), polyethylene glycol (PEG), polyvinylpyrrolidone (PVP)	Fibroblasts	Late infantile neuronal ceroid lipofuscinosis type 2 (LINCL)	LC-3 ↑, Beclin-1 ↑; p62 ↓, LAMP-1 ↑	Transcription factor EB ↑	Autophagy, clearance of lipopigment	[179]
3	Europium hydroxide	None	Neuro 2a, PC12, HeLa	Neuronal and cervical cancer	LC-3 ↑; p62↓	Not reported	Autophagy, clearance of mutant huntingtin	[180]
4	Iron oxide	None	Monocytes	Blood	LC-3 ↑; p62 ↓	Not reported, probably through VEGFR2–PI3K–Akt–mTOR	Autophagy, cell survival	[183]
5	Ferroferric oxide	None	Primary blood cells	Blood	LC-3 ↑; p62↓	Beclin 1/VPS34/Atg14 complex ↑	Autophagy protective	[186]
6	Silica	None	MRC-5	Fibroblasts	LC-3 ↑	ROS dependent	ROS, autophagy protective, cell survival	[188]
7	Iron oxide	lactosylated *N*-alkyl polyethylenimine	Mouse dendritic cells	Immune system	LC-3 ↑	Not reported	Autophagy protective	[189]
8	Bismuth nanoparticles	None	HEK-293	Embryonic kidney	LC-3 ↑, Beclin-1 ↑, ATG12 ↑	PI3K/Akt/mTOR ↓, AMPK ↑	ROS, autophagy protective	[190]
9	Silica	None	HUVEC	Endothelial cells	LC-3 ↑	PI3K/Akt/mTOR ↓	Autophagy, mitophagy, ROS, cell death	[192–194]
10	Iron oxide, titanium oxide, silica	AminoPVA [poly(vinyl alcohol/vinylamine)]	HCECs	Cerebral endothelial cells	LC-3 ↑; p62 ↓	Not reported	ROS, DNA damage, autophagy, lysosome activation	[195]
10	Silica	None	B4G12, HCECs	Corneal endothelial cells	LC-3 ↑	Not reported	Autophagy	[196]
11	Silica	None	BEAS-2B	Bronchial	LC-3 ↑; p62↑	PI3 K/Akt/mTOR↓	ROS, autophagy, cytotoxicity	[197]
12	Silica	None	PC12	Neuronal	LC-3 ↑, Beclin-1 ↑	PI3K/Akt/mTOR↓	Autophagy, ROS, cell death	[198]
13	Iron oxide	None	HUVEC	Endothelial cells	LC-3 ↑, Beclin-1 ↑, VPS43 ↑	Not reported	Autophagy, inflammation	[199]
14	Titanium dioxide	None	HaCaT	Keratinocytes	LC-3 ↑, Beclin-1 ↑, ATG5 ↑	Not reported	Protective autophagy	[207]
15	Zinc oxide	None	BV-2	Microglia	LC-3 ↑, Beclin-1 ↑; p62 ↓	Not reported	Autophagy, mitochondrial damage, ROS, mitophagy, cell death	[208]
16	Copper oxide	dopamine complex	RCSN-3	Neuronal	LC-3 ↑	Not reported	Autophagy, mitophagy, cell death	[209]
17	Copper oxide	None	HUVEC	Endothelial	LC-3 ↑, Beclin-1 ↑, p62 ↑, ATG-5 ↑	Release Cu ions	Autophagy cell death, lysosome dysfunction	[210]
17	Silver	None	Monocytes THP-1	Immune system	LC-3 ↑, p62 ↓	Alkalinisation of lysosomes	Autophagy blockage, lysosome dysfunction, impairment monocyte differentiation	[212]
18	Iron oxide	None	Peritoneal macrophages RAW264.7	Immune system	LC-3 ↑, LAMP-2↑, p62 ↑, ATG-5 ↑	Not reported	ROS, protective autophagy, cell survival	[213]
19	Zinc oxide	None	SupT1, Jurkat; Primary human T-cells;	Immune system	LC-3 ↑, Beclin-1 ↑	Akt/mTOR ↓, release of free Zn^2+^	ROS, exacerbate autophagy, DNA fragmentation, cell death	[214]
20	Zinc oxide	None	Macrophages	Immune system	LC-3 ↑, Beclin-1 ↑	PI3K/Akt/mTOR↓	ROS, autophagy, apoptosis, cell death	[215]
21	Silica	None	Macrophages U937	Immune system	Not reported	Not reported	ROS, IL-8 production, inflammation, Intracellular Ca^2+^ increase	[216]

As occurs for neurodegenerative diseases, also skeletal muscles are often characterized by impaired autophagy clearance and display accumulation of damaged organelles, or misfolded proteins, inside myofibers. Recently, it has been observed that rapamycin-loaded nanoparticles can rescue a correct autophagy flux in *mdx* mice, a model of Duchenne muscular dystrophy, thus increasing skeletal muscle strength that cannot be achieved with pharmacological doses of conventional oral rapamycin. Therefore, rapamycin-loaded nanoparticles could represent an attractive therapeutic alternative by inducing an autophagy clearance in dystrophic muscles [181] (Table 4).

**Table 4:** In vivo assays performed in the studies reported in the review

Entry	NP	Compound carried/combination drug	Mouse model	Disease model	Biological effect	References
1	Silver	Radiotherapy	Orthotopic mouse	Brain cancer	Enhancement in mean survival time, increasing cure rate in glioma-bearing rats	[116]
2	Gold	TRAIL	Nude mice bearing Calu-1 cells	non-small-cell lung cancer (NSCLC)	Reduction tumor growth	[132]
3	Gold	Tmab	Subcutaneous mouse NCI-N87, MKN7	Breast cancer	Growth suppression, autophagy induction	[136]
4	Gold	Quercetin	Old male BALB/c nu/nu nude mice xenograft models	Glioblastoma	Inhibition of tumor growth, low toxicity, improved survival in mice	[140]
5	Gold	Quercetin	Old male BALB/c nu/nu nude mice xenograft models	Cervical cancer	Apoptosis, inhibition cancer growth, and progression	[141]
6	Gold	Poly (acryloyl-l, d and racemic valine)	BALB/C mice and nude mice	Breast cancer	Autophagy, reduction tumor growth	[142]
7	Iron oxide/gold	Anti-EGFR antibody	Old female nude mice	Lung Cancer	Autophagy, DNA damage, apoptosis, tumor growth suppression	[152]
8	Iron oxide	Chitosan chloride (HTCC)/alginate	Gastric SGC7901/ADR^fluc^ tumor-bearing mice	Gastric cancer	Cytotoxicity, autophagy, apoptosis	[153]
9	Iron oxide	Photothermal treatment, CQ	Mude mice bearing MCF-7 xenograft	Breast cancer	Tumor inhibition, autophagosomes accumulation, apoptosis	[157]
10	Cuprous oxide	None	Cervical carcinoma xenograft in nude mice	Cervical cancer	Suppression tumor growth	[162]
11	Rapamycin	None	C57BL10 mice, C57BL/10ScSn-Dmdmdx/J mice	Duchenne muscular dystrophy	Autophagy, recovery of skeletal muscle strength	[181]
12	Silver	None	Male C57BL	Melanoma	Strong cell growth inhibition in combination with autophagy inhibitor	[182]
13	Silver	None	Adult male Sprague–Dawley rats	Liver toxicity	Oxidative stress, markers, hepatotoxicity, protective autophagy	[184]
14	Silica	None	New Zealand white rabbits	Ocular toxicity	Autophagy, no toxicity reported	[196]

Elevation of autophagy level is a common response of cells upon exposure to metallic nanomaterials, and we have summarized the recent studies reporting that a great variety of these nanostructures may induce autophagy cell death in cancer cells. Paradoxically, in some cases it has been reported that metallic nanoparticles may have opposing roles on the cell fate. Emerging evidence indicates that some metallic nanomaterials induce pro-survival autophagy in both cancer and normal cells [120, 156, 182–188]. For example, ferroferric oxide nanoparticles have been shown to induce pro-survival autophagy in human blood cells by modulating the Beclin1/Bcl-2/VPS34 complex [186]. Recently, it has also been reported that lactosylated *N*-alkyl polyethylenimine-coated iron oxide nanoparticles induce protective autophagy in mouse dendritic cells [189]. Bismuth nanoparticles (Bi-NPs) induce protective autophagy in human embryonic kidney cells 293 through the regulation of AMPK/mTOR signal pathway [190]. It has also been published that Ag-NPs induce protective autophagy in HeLa cells by evoking the nuclear translocation of TFEB and consequently the transcription of autophagy and lysosomal-related genes [191].

In all these circumstances, inhibition of autophagy becomes a viable approach for enhancing cancer therapeutic efficacy. However, why some metallic nanomaterials induce pro-death autophagy, while others elicit pro-survival autophagy is poorly understood, and the molecular mechanism underlying these two drastically different effects is largely unexplored.

### Nanotoxicology

Despite the therapeutic advantages of nanomaterials, it is necessary to remind that these products can present some toxicity. Interestingly, the toxicity and the therapeutic effect observed might be derived from the modulation of the autophagy. For instance, Si-NPs have been shown to induce cytotoxicity and autophagy cell death on human umbilical vein, cerebral and corneal endothelial cells through several mechanisms, including ROS generation, dysregulation of PI3K/Akt/mTOR pathway, by affecting angiogenesis and cellular homeostasis, and by leading mitochondrial instability and mitophagy [192–196]. Si-NPs, depending on their size, have also been shown to induce cytotoxicity and autophagy dysfunction in human bronchial epithelial BEAS-2B cells [197]. This occurred through the upregulation of autophagy markers LC3 and p62, and by modulating PI3K/Akt/mTOR pathway in size- and dose-dependent manner [197]. This study shows that Si-NPs could lead autophagy dysfunction and impairment of cellular homeostasis in the respiratory system. Moreover, it has been observed that Si-NPs also may induce autophagy and cell death in neuronal PC12 cells [198]. The autophagy induction, together with ROS increase, and inhibition of ubiquitin–proteasome system (UPS), results in the aggregation of mutant α-synuclein, thus representing a significant risk factor for the development of Parkinson disease [198]. In addition, high concentrations of magnetic iron oxide nanoparticles (Fe_3_O_4_-NPs) have been reported to lead endothelial dysfunction, inflammation and cardiovascular diseases, through both autophagy induction and the blockade of autophagy flux in HUVECs [199]. Titanium dioxide nanoparticles (TiO_2_-NPs) are semiconductor nanomaterials that have been explored for drug delivery purposes and are attracting an increasing level of attention [200, 201]. However, although this nanomaterial has been largely studied for their potent cytotoxic effect in a variety of cancer cells [202–206], it may induce autophagy response in HaCaT human keratinocyte cells [207]. A recent report showed that ZnO-NPs significantly increased the autophagy, ROS level and mitochondrial impairment in BV-2 microglia cell line, in a time-dependent manner. In addition to changes in autophagy markers, a PINK1/parkin-mediated mitophagy has also been reported. The data reported by the authors suggested that mitophagy could play a protective role in ZnO-NP-induced toxicity in BV-2 cells [208]. Despite the therapeutic potential of CO-NPs in a variety of cancer cell lines, a specific neurotoxic action of a copper-dopamine complex in neuronal RCSN-3 cells has also been reported. This occurs by inducing mitochondrial autophagy followed by caspase-3-independent apoptotic cell death [209]. CO-NPs also trigger HUVEC cell death via autophagy and lysosomal dysfunction [210]. It has been reported that polyvinylpyrrolidone (PVP)-coated Ag-NPs have an anti-leukemia effect against human myeloid leukemia cells [182, 185]. However, Ag-NPs have also been shown to trigger cytotoxic autophagy in non-cancer murine pro-B cells (Ba/F3) through the modulation of PI3K/mTOR signaling pathway along with generation of ROS and release of silver ions [117]. Interestingly, some studies report that metallic nanomaterials possess intrinsic toxicity versus immune system components by modulating autophagy. Among the plethora of cells that constitute the immune system, macrophages mediate innate immune responses and contribute to adaptive immunity via antigen processing [211]. It has been reported that Ag-NPs impair monocyte–macrophage differentiation through autophagy blockade, which is mediated by lysosomal dysfunction. Indeed, lysosomal impairment was observed in Ag-NP-treated THP-1 cells, which is responsible for the blockade of autophagic flux [212]. This study suggests a crosstalk among monocyte differentiation, autophagy, and lysosomal dysfunction simultaneously induced by Ag-NPs. In addition, Fe_3_O_4_-NPs have been reported to induce pro-survival autophagy in RAW264.7 cells derived from mouse peritoneal macrophages. The induction of autophagy markers and ROS levels after treatment with Fe_3_O_4_-NPs were accompanied by the ERK pathway that was activated for cell survival [213]. Interestingly, it has been reported that acute exposure to ZnO-NPs induces autophagic immune cell death. This occurs by the release of free Zn(2+) that can be taken up by immune cells triggering the production of excessive intracellular ROS that leads to exacerbated autophagy [214, 215]. Many other studies reported that Si-NPs possess strong toxicity against immune components through the enhancement of proinflammatory responses, oxidative stress and autophagy modulation [216, 217]. These and other studies (reviewed in Peynshaert, 2014) indicate that autophagy modulation mediated by inorganic NPs can potentially represent a risk for immune system, cardiovascular and neurological health. However, the involvement and nature of autophagy deregulation in the pathogenesis of the above-described diseases need further investigation before making conclusions regarding the real cardiovascular and neurological dangers of these nanomaterials and to eventually efficiently target autophagy as a therapeutic strategy.

## Conclusion and future perspective

Human tumors are complex diseases resulting from the interplay between genetic and environmental factors. Besides the many cellular and genetic alterations, cancer cells share common features responsible for their phenotypic manifestations, including uncontrolled proliferation and growth, dysregulation of apoptosis and insensitivity, and severe metabolic alterations [2]. Autophagy is a tightly regulated cellular degradative process by which damaged macromolecules and organelles are targeted by autophagic vesicles to lysosomes and then eliminated [218]. Autophagy is frequently dysregulated in tumors, however, its role in regulating cancer cell death or survival remains highly debated and dependent on metabolic context and on the microenvironmental conditions of the cells [219]. Some studies suggest that basal autophagy may have a protective role in cancer by providing the nutrients necessary for their uncontrolled growth as well as favoring cancer cell survival in many hypoxic tumor microenvironments [48]. However, it is also well assumed that overstimulating autophagy machinery can also lead to cell death, also called cell death-type II, likely due to excessive degradation of cellular constituents and organelles required for homeostasis of the cells [46]. There is mounting evidence that targeting autophagy may be employed as a therapeutic strategy itself or may enhance the efficacy of anticancer therapies [71, 220]. The field of nanotechnology is greatly expanding and can provide the necessary tools to overcome the limits frequently observed with traditional treatments.

This review presents an overview of the most recent reports on NP-mediated autophagy alterations and their impact on nanomedicine. Many studies have shown that nanomaterials and particularly metallic nanoparticles can be used to treat cancer by modulating autophagy. These nanostructures may also promote a plethora of events such as mitochondrial damage, lysosome impairment, ER stress and alterations of signaling pathways, which results in the activation of mitophagy, oxidative stress, and autophagic cell death. Importantly, these materials have shown intrinsic selectivity in inducing autophagy in cancer cells compared to noncancerous cells. However, metal-based nanomaterials may have opposing roles on cell fate being able to induce pro-survival autophagy in cancer and normal cells [162, 184–189]. Thus, inhibition of autophagy may be a viable approach for enhancing cancer therapeutic efficacy.

Autophagy induced by nanomaterials can also be used to treat other diseases such as muscular and neurodegenerative disorders. It is due to their ability to restore a proper autophagic flux, thus removing the protein aggregates and damaged organelles, responsible for the pathogenesis of these diseases [64, 68].

Hence, the capability of many nanostructures to overstimulate autophagy may acquire exceptional medical and toxicological importance. However, more research is needed to define the mechanisms underlying the NP-induced autophagy modulation.

In conclusion, the findings summarized in this review suggest that autophagy modulation with nanoparticle-based strategies would acquire clinical relevance in the near future, as complementary therapies for the treatment of cancers and other diseases.

## References

[1] Stewart BW, Wild CP (2014) World cancer report 2014, World Health Organisation. ISBN-13 978-92-832-0429-9

[2] Hanahan D, Weinberg RA (2000). The hallmarks of cancer. Cancer Immunol Immunother.

[3] Liang XH, Jackson S, Seaman M, Brown K, Kempkes B, Hibshoosh H, Levine B (1999). Induction of autophagy and inhibition of tumorigenesis by beclin 1. Nature.

[4] Mathew R, Karantza-Wadsworth V, White E (2007). Role of autophagy in cancer. Nat Rev Cancer.

[5] Amaravadi RK, Yu D, Lum JJ, Bui T, Christophorou MA, Evan GI, Thomas-Tikhonenko A, Thompson CB (2007). Autophagy inhibition enhances therapy-induced apoptosis in a Myc-induced model of lymphoma. J Clin Investig.

[6] Sinha R (2006). Nanotechnology in cancer therapeutics: bioconjugated nanoparticles for drug delivery. Mol Cancer Ther.

[7] Wicki A, Witzigmann D, Balasubramanian V, Huwyler J (2015). Nanomedicine in cancer therapy: challenges, opportunities, and clinical applications. J Control Release.

[8] Pouponneau P, Leroux JC, Soulez G, Gaboury L, Martel S (2011). Co-encapsulation of magnetic nanoparticles and doxorubicin into biodegradable microcarriers for deep tissue targeting by vascular MRI navigation. Biomaterials.

[9] Abbaszad Rafi A, Mahkam M, Davaran S, Hamishehkar H (2016). A smart pH-responsive nano-carrier as a drug delivery system: a hybrid system comprised of mesoporous nanosilica MCM-41 (as a nano-container) & a pH-sensitive polymer (as smart reversible gatekeepers): preparation, characterization and in vitro release st. Eur J Pharm Sci.

[10] Tran S, DeGiovanni P-J, Piel B, Rai P (2017). Cancer nanomedicine: a review of recent success in drug delivery. Clin Transl Med.

[11] Aftab S, Shah A, Nadhman A, Kurbanoglu S, Aysıl Ozkan S, Dionysiou DD, Shukla SS, Aminabhavi TM (2018). Nanomedicine: an effective tool in cancer therapy. Int J Pharm.

[12] Bobo D, Robinson KJ, Islam J, Thurecht KJ, Corrie SR (2016). Nanoparticle-based medicines: a review of FDA-approved materials and clinical trials to date. Pharm Res.

[13] Wang YC, Wang F, Sun TM, Wang J (2011). Redox-responsive nanoparticles from the single disulfide bond-bridged block copolymer as drug carriers for overcoming multidrug resistance in cancer cells. Bioconj Chem.

[14] Panzarini E, Dini L (2014). Nanomaterial-induced autophagy: a new reversal MDR tool in cancer therapy?. Mol Pharm.

[15] Peynshaert K, Manshian BB, Joris F, Braeckmans K, De Smedt SC, Demeester J, Soenen SJ (2014). Exploiting intrinsic nanoparticle toxicity: the pros and cons of nanoparticle-induced autophagy in biomedical research. Chem Rev.

[16] Mizushima N (2009). Physiological functions of autophagy. Curr Top Microbiol Immunol.

[17] Mizushima N (2007). Autophagy: process and function. Genes Dev.

[18] Pattingre S, Espert L, Biard-Piechaczyk M, Codogno P (2008). Regulation of macroautophagy by mTOR and Beclin 1 complexes. Biochimie.

[19] Backer JM (2008). The regulation and function of Class III PI3Ks: novel roles for Vps34. Biochem J.

[20] Øvervatn A, Bjørkøy G, Johansen T (2007). p62/SQSTM1 binds directly to Atg8/LC3 to facilitate degradation of ubiquitinated protein aggregates. J Biol Chem.

[21] Kirkin V, McEwan DG, Novak I, Dikic I (2009). A role for ubiquitin in selective autophagy. Mol Cell.

[22] Gwinn DM, Shackelford DB, Egan DF, Mihaylova MM, Mery A, Vasquez DS, Turk BE, Shaw RJ (2008). AMPK phosphorylation of raptor mediates a metabolic checkpoint. Mol Cell.

[23] Inoki K, Ouyang H, Zhu T, Lindvall C, Wang Y, Zhang X, Yang Q, Bennett C, Harada Y, Stankunas K, yu Wang C, He X, MacDougald OA, You M, Williams BO, Guan KL (2006). TSC2 integrates Wnt and energy signals via a coordinated phosphorylation by AMPK and GSK3 to regulate cell growth. Cell.

[24] Kim J, Kundu M, Viollet B, Guan KL (2011). AMPK and mTOR regulate autophagy through direct phosphorylation of Ulk1. Nat Cell Biol.

[25] Noda T, Ohsumi Y (1998). Tor, a phosphatidylinositol kinase homologue, controls autophagy in yeast. J Biol Chem.

[26] Wullschleger S, Loewith R, Hall MN (2006). TOR signaling in growth and metabolism. Cell.

[27] Arias E, Koga H, Diaz A, Mocholi E, Patel B, Cuervo AM (2015). Lysosomal mTORC2/PHLPP1/Akt regulate chaperone-mediated autophagy. Mol Cell.

[28] Mammucari C, Milan G, Romanello V, Masiero E, Rudolf R, Del Piccolo P, Burden SJ, Di Lisi R, Sandri C, Zhao J, Goldberg AL, Schiaffino S, Sandri M (2007). FoxO3 controls autophagy in skeletal muscle in vivo. Cell Metab.

[29] Mizushima N (2010). The role of the Atg1/ULK1 complex in autophagy regulation. Curr Opin Cell Biol.

[30] Crighton D, Wilkinson S, Ryan KM (2007). DRAM links autophagy to p53 and programmed cell death. Autophagy.

[31] Jones RG, Plas DR, Kubek S, Buzzai M, Mu J, Xu Y, Birnbaum MJ, Thompson CB (2005). AMP-activated protein kinase induces a p53-dependent metabolic checkpoint. Mol Cell.

[32] Okoshi R, Ozaki T, Yamamoto H, Ando K, Koida N, Ono S, Koda T, Kamijo T, Nakagawara A, Kizaki H (2008). Activation of AMP-activated protein kinase induces p53-dependent apoptotic cell death in response to energetic stress. J Biol Chem.

[33] He G, Zhang Y-W, Lee J-H, Zeng SX, Wang YV, Luo Z, Dong XC, Viollet B, Wahl GM, Lu H (2014). AMP-activated protein kinase induces p53 by phosphorylating MDMX and inhibiting its activity. Mol Cell Biol.

[34] Feng Z, Hu W, De Stanchina E, Teresky AK, Jin S, Lowe S, Levine AJ (2007). The regulation of AMPK β1, TSC2, and PTEN expression by p53: stress, cell and tissue specificity, and the role of these gene products in modulating the IGF-1-AKT-mTOR pathways. Cancer Res.

[35] Budanov AV, Karin M (2008). p53 target genes sestrin1 and sestrin2 connect genotoxic stress and mTOR signaling. Cell.

[36] Vyas S, Zaganjor E, Haigis MC (2016). Mitochondria and cancer. Cell.

[37] Eiyama A, Okamoto K (2015). PINK1/Parkin-mediated mitophagy in mammalian cells. Curr Opin Cell Biol.

[38] Ashrafi G, Schwarz TL (2013). The pathways of mitophagy for quality control and clearance of mitochondria. Cell Death Differ.

[39] Kanki T, Wang K, Baba M, Bartholomew CR, Lynch-Day MA, Du Z, Geng J, Mao K, Yang Z, Yen W-L, Klionsky DJ (2009). A genomic screen for yeast mutants defective in selective mitochondria autophagy. Mol Biol Cell.

[40] Redmann M, Dodson M, Boyer-Guittaut M, Darley-Usmar V, Zhang J (2014). Mitophagy mechanisms and role in human diseases. Int J Biochem Cell Biol.

[41] Chourasia AH, Tracy K, Frankenberger C, Boland ML, Sharifi MN, Drake LE, Sachleben JR, Asara JM, Locasale JW, Karczmar GS, Macleod KF (2015). Mitophagy defects arising from BNip3 loss promote mammary tumor progression to metastasis. EMBO Rep.

[42] Yan C, Luo L, Guo CY, Goto S, Urata Y, Shao JH, Li TS (2017). Doxorubicin-induced mitophagy contributes to drug resistance in cancer stem cells from HCT8 human colorectal cancer cells. Cancer Lett.

[43] Shintani T, Klionsky DJ (2004). Autophagy in health and disease: A double-edged sword. Science (80-.).

[44] Dodson M, Darley-Usmar V, Zhang J (2013). Cellular metabolic and autophagic pathways: traffic control by redox signaling. Free Radic Biol Med.

[45] Deberardinis RJ, Thompson CB (2012). Cellular metabolism and disease: what do metabolic outliers teach us?. Cell.

[46] Kondo Y, Kanzawa T, Sawaya R, Kondo S (2005). The role of autophagy in cancer development and response to therapy. Nat Rev Cancer.

[47] Singh SS, Vats S, Chia AY-Q, Tan TZ, Deng S, Ong MS, Arfuso F, Yap CT, Goh BC, Sethi G, Huang RY-J, Shen HM, Manjithaya R, Kumar AP (2018). Dual role of autophagy in hallmarks of cancer. Oncogene.

[48] Fiorini C, Menegazzi M, Padroni C, Dando I, Dalla Pozza E, Gregorelli A, Costanzo C, Palmieri M, Donadelli M (2013). Autophagy induced by p53-reactivating molecules protects pancreatic cancer cells from apoptosis. Apoptosis.

[49] Bellot G, Garcia-Medina R, Gounon P, Chiche J, Roux D, Pouyssegur J, Mazure NM (2009). Hypoxia-induced autophagy is mediated through hypoxia-inducible factor induction of BNIP3 and BNIP3L via their BH3 domains. Mol Cell Biol.

[50] Kimmelman AC (2011). The dynamic nature of autophagy in cancer. Genes Dev.

[51] Yang ZJ, Chee CE, Huang S, Sinicrope FA (2011). The role of autophagy in cancer: therapeutic implications. Mol Cancer Ther.

[52] Kumari S, Badana AK, Murali MG, Shailender G, Malla R (2018) Reactive oxygen species: a key constituent in cancer survival, biomark. Insights 13:117727191875539. 10.1177/1177271918755391 10.1177/1177271918755391 PMC5808965 29449774

[53] Mathew R, Kongara S, Beaudoin B, Karp CM, Bray K, Degenhardt K, Chen G, Jin S, White E (2007). Chromosomal instability autophagy suppresses tumor progression by limiting chromosomal instability. Genes Dev.

[54] White E (2012). Deconvoluting the context-dependent role for autophagy in cancer. Nat Rev Cancer.

[55] Shen Y, Li DD, Wang LL, Deng R, Zhu XF (2008). Decreased expression of autophagy-related proteins in malignant epithelial ovarian cancer. Autophagy.

[56] Moscat J, Diaz-Meco MT (2009). p62 at the crossroads of autophagy, apoptosis, and cancer. Cell.

[57] Liu J, Xia H, Kim M, Xu L, Li Y, Zhang L, Cai Y, Norberg HV, Zhang T, Furuya T, Jin M, Zhu Z, Wang H, Yu J, Li Y, Hao Y, Choi A, Ke H, Ma D, Yuan J (2011). Beclin1 controls the levels of p53 by regulating the deubiquitination activity of USP10 and USP13. Cell.

[58] Agarwal S, Bell CM, Taylor SM, Moran RG (2016). p53 deletion or hotspot mutations enhance mTORC1 activity by altering lysosomal dynamics of TSC2 and Rheb. Mol Cancer Res.

[59] Tan BS, Tiong KH, Choo HL, Fei-Lei Chung F, Hii LW, Tan SH, Yap IKS, Pani S, Khor NTW, Wong SF, Rosli R, Cheong SK, Leong CO (2015). Mutant p53-R273H mediates cancer cell survival and anoikis resistance through AKT-dependent suppression of BCL2-modifying factor (BMF). Cell Death Dis.

[60] Cordani M, Oppici E, Dando I, Butturini E, Dalla Pozza E, Nadal-Serrano M, Oliver J, Roca P, Mariotto S, Cellini B, Blandino G, Palmieri M, Di Agostino S, Donadelli M (2016). Mutant p53 proteins counteract autophagic mechanism sensitizing cancer cells to mTOR inhibition. Mol Oncol.

[61] Zhou G, Wang J, Zhao M, Xie TX, Tanaka N, Sano D, Patel AA, Ward AM, Sandulache VC, Jasser SA, Skinner HD, Fitzgerald AL, Osman AA, Wei Y, Xia X, Songyang Z, Mills GB, Hung MC, Caulin C, Liang J, Myers JN (2014). Gain-of-function mutant p53 promotes cell growth and cancer cell metabolism via inhibition of AMPK activation. Mol Cell.

[62] Cordani M, Butera G, Pacchiana R, Donadelli M (1867). Molecular interplay between mutant p53 proteins and autophagy in cancer cells. Biochim Biophys Acta Rev Cancer.

[63] Popovic D, Vucic D, Dikic I (2014). Ubiquitination in disease pathogenesis and treatment. Nat Med.

[64] Martinez-Vicente M, Cuervo AM (2007). Autophagy and neurodegeneration: when the cleaning crew goes on strike. Lancet Neurol.

[65] Sandri M (2011). New findings of lysosomal proteolysis in skeletal muscle. Curr Opin Clin Nutr Metab Care.

[66] Nishino I, Fu J, Tanji K, Yamada T, Shimojo S, Koori T, Mora M, Riggs JE, Oh SJ, Koga Y, Sue CM, Yamamoto A, Murakami N, Shanske S, Byrne E, Bonilla E, Honaka I, DiMauro S, Hirano M (2000). Primary LAMP-2 deficiency causes X-linked vacoular cardiomyopathy and myopathy (Danon disease). Nature.

[67] Fukuda T, Ahearn M, Roberts A, Mattaliano RJ, Zaal K, Ralston E, Plotz PH, Raben N (2006). Autophagy and mistargeting of therapeutic enzyme in skeletal muscle in Pompe disease. Mol Ther.

[68] Sandri M, Coletto L, Grumati P, Bonaldo P (2013). Misregulation of autophagy and protein degradation systems in myopathies and muscular dystrophies. J Cell Sci.

[69] Hu Y-L, DeLay M, Jahangiri A, Molinaro AM, Rose SD, Carbonell WS, Aghi MK (2012). Hypoxia-induced autophagy promotes tumor cell survival and adaptation to antiangiogenic treatment in glioblastoma. Cancer Res.

[70] Kenific CM, Thorburn A, Debnath J (2010). Autophagy and metastasis: another double-edged sword. Curr Opin Cell Biol.

[71] Duffy A, Le J, Sausville E, Emadi A (2015). Autophagy modulation: a target for cancer treatment development. Cancer Chemother Pharmacol.

[72] Cook KL, Shajahan AN, Clarke R (2011). Autophagy and endocrine resistance in breast cancer. Expert Rev Anticancer Ther.

[73] Jain K, Paranandi KS, Sridharan S, Basu A (2013). Autophagy in breast cancer and its implications for therapy. Am J Cancer Res.

[74] Jin S, White E (2007). Role of autophagy in cancer: management of metabolic stress. Autophagy.

[75] Sui X, Chen R, Wang Z, Huang Z, Kong N, Zhang M, Han W, Lou F, Yang J, Zhang Q, Wang X, He C, Pan H (2013). Autophagy and chemotherapy resistance: a promising therapeutic target for cancer treatment. Cell Death Dis.

[76] Ronan B, Flamand O, Vescovi L, Dureuil C, Durand L, Fassy F, Bachelot MF, Lamberton A, Mathieu M, Bertrand T, Marquette JP, El-Ahmad Y, Filoche-Romme B, Schio L, Garcia-Echeverria C, Goulaouic H, Pasquier B, Thoreen CC, Kang SA, Chang JW, Liu Q, Zhang J, Gao Y, Reichling LJ, Sim T, Sabatini DM, Gray NS, Taniguchi M, Kitatani K, Kondo T, Hashimoto-Nishimura M, Asano S, Hayashi A, Mitsutake S, Igarashi Y, Umehara H, Takeya H, Kigawa J, Okazaki T, Ponpuak M, Mandell MA, Kimura T, Chauhan S, Cleyrat C, Deretic V, Cell IF, Glucose W, Kumar A, Lawrence JC, Jung DY, Ko HJ, Keller SR, Kim JK, Magnuson MA, Harris TE, Torres-quiroz F, Filteau M, Landry CR, Dowdle WE, Nyfeler B, Nagel J, Elling RA, Liu S, Triantafellow E, Menon S, Wang Z, Honda A, Pardee G, Cantwell J, Luu C, Cornella-Taracido I, Harrington E, Fekkes P, Lei H, Fang Q, Digan ME, Burdick D, Powers AF, Helliwell SB, Daquin S, Bastien J, Wang H, Wiederschain D, Kuerth J, Bergman P, Schwalb D, Thomas J, Ugwonali S, Harbinski F, Tallarico J, Wilson CJ, Myer VE, Porter JA, Bussiere DE, Finan PM, Labow MA, Mao X, Hamann LG, Manning BD, Valdez RA, Nicholson T, Schirle M, Knapp MS, Keaney EP, Murphy LO, Soliman GA, Acosta-Jaquez HA, Fingar DC, Sarkar S, Mizushima N, Yoshimorim T, Levine B, Wu YT, Tan HL, Shui G, Bauvy C, Huang Q, Wenk MR, Ong CN, Codogno P, Shen HM, Yang YP, Hu LF, Zheng HF, Mao CJ, Hu WD, Xiong KP, Wang F, Liu CF, Committee E, Grijalva A, Xu X, Ferrante AW, Ugland H, Naderi S, Brech A, Collas P, Blomhoff HK, Chen Y, Klionsky DJ, Dupont N, Jiang S, Pilli M, Ornatowski W, Bhattacharya D, Deretic V, Alers S, Löffler AS, Paasch F, Dieterle AM, Keppeler H, Lauber K, Campbell DG, Fehrenbacher B, Schaller M, Wesselborg S, Stork B, Popelka H, Klionsky DJ, Sun K, Kusminski CCM, Scherer PEP, Humphrey SJ, Yang G, Yang P, Fazakerley DJ, Stöckli J, Yang JY, James DE, Heckmann BL, Yang X, Zhang X, Liu J, Goold R, McKinnon C, Rabbanian S, Collinge J, Schiavo G, Tabrizi SJ, Manjithaya R, Subramani S (2011). Application and interpretation of current autophagy inhibitors and activators. Autophagy.

[77] Goldberg SB, Supko JG, Neal JW, Muzikansky A, Digumarthy S, Fidias P, Temel JS, Heist RS, Shaw AT, McCarthy PO, Lynch TJ, Sharma S, Settleman JE, Sequist LV (2012). A phase I study of erlotinib and hydroxychloroquine in advanced non-small-cell lung cancer. J Thorac Oncol.

[78] Lee HO, Mustafa A, Hudes GR, Kruger WD (2015). Hydroxychloroquine destabilizes phospho-S6 in human renal carcinoma cells. PLoS One.

[79] Cook KL, Wärri A, Soto-Pantoja DR, Clarke PAG, Cruz MI, Zwart A, Clarke R (2014). Hydroxychloroquine inhibits autophagy to potentiate antiestrogen responsiveness in ER+ breast cancer. Clin Cancer Res.

[80] Milano V, Piao Y, LaFortune T, de Groot J (2009). Dasatinib-induced autophagy is enhanced in combination with temozolomide in glioma. Mol Cancer Ther.

[81] Liu YL, Yang PM, Shun CT, Wu MS, Weng JR, Chen CC (2010). Autophagy potentiates the anti-cancer effects of the histone deacetylase inhibitors in hepatocellular carcinoma. Autophagy.

[82] Zhu K, Dunner K, McConkey DJ (2010). Proteasome inhibitors activate autophagy as a cytoprotective response in human prostate cancer cells. Oncogene.

[83] Yazbeck VY, Buglio D, Georgakis GV, Li Y, Iwado E, Romaguera JE, Kondo S, Younes A (2008). Temsirolimus downregulates p21 without altering cyclin D1 expression and induces autophagy and synergizes with vorinostat in mantle cell lymphoma. Exp Hematol.

[84] Crazzolara R, Cisterne A, Thien M, Hewson J, Baraz R, Bradstock KF, Bendall LJ (2009). Potentiating effects of RAD001 (Everolimus) on vincristine therapy in childhood acute lymphoblastic leukemia. Blood.

[85] Guba M, Von Breitenbuch P, Steinbauer M, Koehl G, Flegel S, Hornung M, Bruns CJ, Zuelke C, Farkas S, Anthuber M, Jauch KW, Geissler EK (2002). Rapamycin inhibits primary and metastatic tumor growth by antiangiogenesis: involvement of vascular endothelial growth factor. Nat Med.

[86] Motzer RJ, Escudier B, Oudard S, Hutson TE, Porta C, Bracarda S, Grünwald V, Thompson JA, Figlin RA, Hollaender N, Urbanowitz G, Berg WJ, Kay A, Lebwohl D, Ravaud A (2008). Efficacy of everolimus in advanced renal cell carcinoma: a double-blind, randomised, placebo-controlled phase III trial. Lancet.

[87] Xie X, White EP, Mehnert JM (2013). Coordinate autophagy and mTOR pathway inhibition enhances cell death in melanoma. PLoS One.

[88] Rosich L, Xargay-Torrent S, López-Guerra M, Campo E, Colomer D, Roué G (2012). Counteracting autophagy overcomes resistance to everolimus in mantle cell lymphoma. Clin Cancer Res.

[89] Conde J, Doria G, Baptista P (2012). Noble metal nanoparticles applications in cancer. J Drug Deliv.

[90] Sun T, Zhang YS, Pang B, Hyun DC, Yang M, Xia Y (2014). Engineered nanoparticles for drug delivery in cancer therapy. Angew Chem Int Ed.

[91] Hong H, Shi J, Yang Y, Zhang Y, Engle JW, Nickles RJ, Wang X, Cai W (2011). Cancer-targeted optical imaging with fluorescent zinc oxide nanowires. Nano Lett.

[92] Pagliari F, Mandoli C, Forte G, Magnani E, Pagliari S, Nardone G, Licoccia S, Minieri M, Di Nardo P, Traversa E (2012). Cerium oxide nanoparticles protect cardiac progenitor cells from oxidative stress. ACS Nano.

[93] Partha R, Mitchell LR, Lyon JL, Joshi PP, Conyers JL (2008). Buckysomes: fullerene-based nanocarriers for hydrophobic molecule delivery. ACS Nano.

[94] Yang W, Thordarson P, Gooding JJ, Ringer SP, Braet F (2001). Carbon nanotubes for biological and biomedical applications.

[95] Williams KA, Veenhuizen PTM, De la Torre BG, Eritja R, Dekker C (2002). Nanotechnology: carbon nanotubes with DNA recognition. Nature.

[96] Matsumura Y, Kataoka K (2009). Preclinical and clinical studies of anticancer agent-incorporating polymer micelles. Cancer Sci.

[97] Dreaden EC, Alkilany AM, Huang X, Murphy CJ, El-Sayed MA (2012). The golden age: gold nanoparticles for biomedicine. Chem Soc Rev.

[98] Bhattacharya R, Mukherjee P (2008). Biological properties of “naked” metal nanoparticles. Adv Drug Deliv Rev.

[99] Jain S, Hirst DG, O’Sullivan JM (2012). Gold nanoparticles as novel agents for cancer therapy. Br J Radiol.

[100] Albanese A, Tang PS, Chan WCW (2012). The effect of nanoparticle size, shape, and surface chemistry on biological systems. Annu Rev Biomed Eng.

[101] Liu Y, Solomon M, Achilefu S (2013). Perspectives and potential applications of nanomedicine in breast and prostate cancer. Med Res Rev.

[102] Cho K, Wang X, Nie S, Chen ZG, Shin DM (2008). Therapeutic nanoparticles for drug delivery in cancer. Clin Cancer Res.

[103] Bañobre-López M, Teijeiro A, Rivas J (2013). Magnetic nanoparticle-based hyperthermia for cancer treatment. Rep Pract Oncol Radiother.

[104] Andrä W, Nowak H (2006) Magnetism in Medicine: a Handbook, 2nd edn. WILEY-VCH Verlag GmbH & Co. KGaA, Weinheim. 10.1002/9783527610174

[105] Giustini AJ, Petryk AA, Cassim SM, Tate JA, Baker I, Hoopes PJ (2010). Magnetic nanoparticle hyperthermia in cancer treatment. Nano Life.

[106] Huang X, Jain PK, El-Sayed IH, El-Sayed MA (2008). Plasmonic photothermal therapy (PPTT) using gold nanoparticles. Lasers Med Sci.

[107] Chu M, Shao Y, Peng J, Dai X, Li H, Wu Q, Shi D (2013). Near-infrared laser light mediated cancer therapy by photothermal effect of Fe_3_O_4_ magnetic nanoparticles. Biomaterials.

[108] Bernardi RJ, Lowery AR, Thompson PA, Blaney SM, West JL (2008). Immunonanoshells for targeted photothermal ablation in medulloblastoma and glioma: an in vitro evaluation using human cell lines. J Neurooncol.

[109] Cheng FY, Chen CT, Yeh CS (2009). Comparative efficiencies of photothermal destruction of malignant cells using antibody-coated silica@Au nanoshells, hollow Au/Ag nanospheres and Au nanorods. Nanotechnology.

[110] Dickerson EB, Dreaden EC, Huang X, El-Sayed IH, Chu H, Pushpanketh S, McDonald JF, El-Sayed MA (2008). Gold nanorod assisted near-infrared plasmonic photothermal therapy (PPTT) of squamous cell carcinoma in mice. Cancer Lett.

[111] Diagaradjane P, Shetty A, Wang JC, Elliott AM, Schwartz J, Shentu S, Park HC, Deorukhkar A, Stafford RJ, Cho SH, Tunnell JW, Hazle JD, Krishnan S (2008). Modulation of in vivo tumor radiation response via gold nanoshell-mediated vascular-focused hyperthermia: characterizing an integrated antihypoxic and localized vascular disrupting targeting strategy. Nano Lett.

[112] Buttacavoli M, Albanese NN, Di Cara G, Alduina R, Faleri C, Gallo M, Pizzolanti G, Gallo G, Feo S, Baldi F, Cancemi P (2018). Anticancer activity of biogenerated silver nanoparticles: an integrated proteomic investigation. Oncotarget..

[113] Zielinska E, Zauszkiewicz-pawlak A, Wojcik M (2017). Silver nanoparticles of different sizes induce a mixed type of programmed cell death in human pancreatic ductal adenocarcinoma. Oncotarget.

[114] Yuan YG, Gurunathan S (2017). Combination of graphene oxide-silver nanoparticle nanocomposites and cisplatin enhances apoptosis and autophagy in human cervical cancer cells. Int J Nanomed.

[115] Zhang X-F, Gurunathan S (2016). Combination of salinomycin and silver nanoparticles enhances apoptosis and autophagy in human ovarian cancer cells: an effective anticancer therapy. Int J Nanomed.

[116] Liu P, Jin H, Guo Z, Ma J, Zhao J, Li D, Wu H, Gu N (2016). Silver nanoparticles outperform gold nanoparticles in radiosensitizing U251 cells in vitro and in an intracranial mouse model of glioma. Int J Nanomed.

[117] Zhu L, Guo D, Sun L, Huang Z, Zhang X, Ma W, Wu J, Xiao L, Zhao Y, Gu N (2017). Activation of autophagy by elevated reactive oxygen species rather than released silver ions promotes cytotoxicity of polyvinylpyrrolidone-coated silver nanoparticles in hematopoietic cells. Nanoscale.

[118] Mishra AR, Zheng J, Tang X, Goering PL (2016). Silver nanoparticle-induced autophagic-Lysosomal disruption and NLRP3-inflammasome activation in HepG2 cells is size-dependent. Toxicol Sci.

[119] Jeong JK, Gurunathan S, Kang MH, Han JW, Das J, Choi YJ, Kwon DN, Cho SG, Park C, Seo HG, Song H, Kim JH (2016). Hypoxia-mediated autophagic flux inhibits silver nanoparticle-triggered apoptosis in human lung cancer cells. Sci Rep.

[120] Fageria L, Pareek V, Dilip RV, Bhargava A, Pasha SS, Laskar IR, Saini H, Dash S, Chowdhury R, Panwar J (2017). Biosynthesized protein-capped silver nanoparticles induce ROS-dependent proapoptotic signals and prosurvival autophagy in cancer cells. ACS Omega.

[121] Settembre C, Di Malta C, Polito VA, Arencibia MG, Vetrini F, Erdin S, Erdin SU, Huynh T, Medina D, Colella P, Sardiello M, Rubinsztein DC, Ballabio A (2011). TFEB links autophagy to lysosomal biogenesis. Science (80-.).

[122] Settembre C, Ballabio A (2011). TFEB regulates autophagy: an integrated coordination of cellular degradation and recycling processes. Autophagy.

[123] Miyayama T, Fujiki K, Matsuoka M (2018). Silver nanoparticles induce lysosomal-autophagic defects and decreased expression of transcription factor EB in A549 human lung adenocarcinoma cells. Toxicol Vitro.

[124] Eck W, Craig G, Sigdel A, Ritter G, Old LJ, Tang L, Brennan MF, Allen PJ, Mason MD (2008). PEGylated gold nanoparticles conjugated to monoclonal F19 antibodies as targeted labeling agents for human pancreatic carcinoma tissue. ACS Nano.

[125] Connor EE, Mwamuka J, Gole A, Murphy CJ, Wyatt MD (2005). Gold nanoparticles are taken up by human cells but do not cause acute cytotoxicity. Small.

[126] Klassen NV, Kedrov VV, Ossipyan YA, Shmurak SZ, Shmytńko IM, Krivko OA, Kudrenko EA, Kurlov VN, Kobelev NP, Kiselev AP, Bozhko SI (2009). Nanoscintillators for microscopic diagnostics of biological and medical objects and medical therapy. IEEE Trans Nanobiosci.

[127] Lin YX, Gao YJ, Wang Y, Qiao ZY, Fan G, Qiao SL, Zhang RX, Wang L, Wang H (2015). pH-sensitive polymeric nanoparticles with gold(I) compound payloads synergistically induce cancer cell death through modulation of autophagy. Mol Pharm.

[128] Koken MHM, Smit EME, Jaspers-Dekker I, Oostra BA, Hagemeuer A, Bootsma D, Hoeumakers JHJ (1992). Localization of two human homologs, HHR6A and HHR6B, of the yeast DNA repair gene RAD6 to chromosomes Xq24-q25 and 5q23-q31. Genomics.

[129] Koken MH, Reynolds P, Jaspers-Dekker I, Prakash L, Prakash S, Bootsma D, Hoeijmakers JH (1991). Structural and functional conservation of two human homologs of the yeast DNA repair gene RAD6. Proc Natl Acad Sci USA.

[130] Haynes B, Zhang Y, Liu F, Li J, Petit S, Bao X, Westwell AD, Mao G, Building R (2016). Gold nanoparticle conjugated Rad6 inhibitor induces cell death in triple negative breast cancer cells by inducing mitochondrial dysfunction and PARP-1 hyperactivation: synthesis and characterization. Nanomedicine.

[131] Bhowmik T, Gomes A (2016). NKCT1 (purified Naja kaouthia protein toxin) conjugated gold nanoparticles induced Akt/mTOR inactivation mediated autophagic and caspase 3 activated apoptotic cell death in leukemic cell. Toxicon.

[132] Ke S, Zhou T, Yang P, Wang Y, Zhang P, Chen K, Ren L, Ye S (2017). Gold nanoparticles enhance TRAIL sensitivity through Drp1-mediated apoptotic and autophagic mitochondrial fission in NSCLC cells. Int J Nanomed.

[133] Crown J, O’Shaughnessy J, Gullo G (2012). Emerging targeted therapies in triple-negative breast cancer. Ann Oncol.

[134] Zhang M, Kim HS, Jin T, Moon WK (2017). Near-infrared photothermal therapy using EGFR-targeted gold nanoparticles increases autophagic cell death in breast cancer. J Photochem Photobiol B Biol.

[135] Slamon DJ, Leyland-Jones B, Shak S, Fuchs H, Paton V, Bajamonde A, Fleming T, Eiermann W, Wolter J, Pegram M, Baselga J, Norton L (2001). Use of chemotherapy plus a monoclonal antibody against HER2 for metastatic breast cancer that overexpresses HER2. N Engl J Med.

[136] Kubota T, Kuroda S, Kanaya N, Morihiro T, Aoyama K, Yoshihiko K, Kikuchi S, Nishizaki M, Kagawa S, Tazawa H, Fujiwara T (2018). HER2-targeted gold nanoparticles potentially overcome resistance to trastuzumab in gastric cancer. Nanomed Nanotechnol Biol Med.

[137] Formica JV, Regelson W (1995). Review of the biology of quercetin and related bioflavonoids. Food Chem Toxicol.

[138] Rauf A, Imran M, Khan IA, ur-Rehman M, Gilani SA, Mehmood Z, Mubarak MS (2018). Anticancer potential of quercetin: a comprehensive review. Phyther Res.

[139] Ren K-W, Li Y-H, Wu G, Ren J-Z, Lu H-B, Li Z-M, Han X-W (2017). Quercetin nanoparticles display antitumor activity via proliferation inhibition and apoptosis induction in liver cancer cells. Int J Oncol.

[140] Lou M, Na Zhang L, Gang Ji P, Qiang Feng F, Hui Liu J, Yang C, Fu Li B, Wang L (2016). Quercetin nanoparticles induced autophagy and apoptosis through AKT/ERK/caspase-3 signaling pathway in human neuroglioma cells: in vitro and in vivo. Biomed Pharmacother.

[141] Lin Luo C, Qiong Liu Y, Wang P, Hua Song C, Juan Wang K, Ping Dai L, Ying Zhang J, Ye H (2016). The effect of quercetin nanoparticle on cervical cancer progression by inducing apoptosis, autophagy and anti-proliferation via JAK2 suppression. Biomed Pharmacother.

[142] Yuan L, Zhang F, Qi X, Yang Y, Yan C, Jiang J, Deng J (2018). Chiral polymer modified nanoparticles selectively induce autophagy of cancer cells for tumor ablation. J Nanobiotechnol.

[143] Carpenter EE, Sangregorio C, Connor CJ (1999). Effects of shell thickness on blocking temperature of nanocomposites of metal particles with gold shells. IEEE Trans Magn.

[144] Wu YN, Yang LX, Shi XY, Li IC, Biazik JM, Ratinac KR, Chen DH, Thordarson P, Bin Shieh D, Braet F (2011). The selective growth inhibition of oral cancer by iron core-gold shell nanoparticles through mitochondria-mediated autophagy. Biomaterials.

[145] Wu Y-N, Wu P-C, Yang L-X, Ratinac KR, Thordarson P, Jahn KA, Chen D-H, Shieh D-B, Braet F (2013). The anticancer properties of iron core–gold shell nanoparticles in colorectal cancer cells. Int J Nanomed.

[146] Rasmussen JW, Martinez E, Louka P, Wingett DG (2010). Zinc oxide nanoparticles for selective destruction of tumor cells and potential for drug delivery applications. Expert Opin Drug Deliv.

[147] Bai D-P, Zhang X-F, Zhang G-L, Huang Y-F, Gurunathan S (2017). Zinc oxide nanoparticles induce apoptosis and autophagy in human ovarian cancer cells. Int J Nanomed.

[148] Wang J, Gao S, Wang S, Xu Z, Wei L (2018). Zinc oxide nanoparticles induce toxicity in CAL 27 oral cancer cell lines by activating PINK1/Parkin-mediated mitophagy. Int J Nanomed.

[149] Mozdoori N, Safarian S, Sheibani N (2017). Augmentation of the cytotoxic effects of zinc oxide nanoparticles by MTCP conjugation: non-canonical apoptosis and autophagy induction in human adenocarcinoma breast cancer cell lines. Mater Sci Eng C.

[150] Yang C, He X, Song L, Zhan X, Zhang Y, Dou J, Gu N (2014). Gamma-Fe_2_O_3_ nanoparticles increase therapeutic efficacy of combination with paclitaxel and anti-ABCG2 monoclonal antibody on multiple myeloma cancer stem cells in mouse model. J Biomed Nanotechnol.

[151] Zhang X, Zhang H, Liang X, Zhang J, Tao W, Zhu X, Chang D, Zeng X, Liu G, Mei L (2016). Iron oxide nanoparticles induce autophagosome accumulation through multiple mechanisms: lysosome impairment, mitochondrial damage, and ER stress. Mol Pharm.

[152] Kuroda S, Tam J, Roth JA, Sokolov K, Ramesh R (2014). EGFR-targeted plasmonic magnetic nanoparticles suppress lung tumor growth by abrogating G2/M cell-cycle arrest and inducing DNA damage. Int J Nanomed.

[153] Li X, Feng J, Zhang R, Wang J, Su T, Tian Z, Han D, Zhao C, Fan M, Li C, Liu B, Feng X, Nie Y, Wu K, Chen Y, Deng H, Feng C (2016). Quaternized chitosan/alginate-Fe_3_O_4_ magnetic nanoparticles enhance the chemosensitization of multidrug-resistant gastric carcinoma by regulating cell autophagy activity in mice. J Biomed Nanotechnol.

[154] Khan MI, Mohammad A, Patil G, Naqvi SAH, Chauhan LKS, Ahmad I (2012). Induction of ROS, mitochondrial damage and autophagy in lung epithelial cancer cells by iron oxide nanoparticles. Biomaterials.

[155] Huang D, Zhou H, Gao J (2015). Nanoparticles modulate autophagic effect in a dispersity-dependent manner. Sci Rep.

[156] Feng Q, Liu Y, Huang J, Chen K, Huang J, Xiao K (2018). Uptake, distribution, clearance, and toxicity of iron oxide nanoparticles with different sizes and coatings. Sci Rep.

[157] Ren X, Chen Y, Peng H, Fang X, Zhang X, Chen Q, Wang X, Yang W, Sha X (2018). Blocking autophagic flux enhances iron oxide nanoparticles photothermal therapeutic efficiency in cancer treatment. ACS Appl Mater Interfaces.

[158] Wang Y, Zi X-Y, Su J, Zhang H-X, Zhang X-R, Zhu H-Y, Li J-X, Yin M, Yang F, Hu Y-P (2012). Cuprous oxide nanoparticles selectively induce apoptosis of tumor cells. Int J Nanomed.

[159] Wang Y, Yang F, Zhang H-X, Zi X-Y, Pan X-H, Chen F, Luo W-D, Li J-X, Zhu H-Y, Hu Y-P (2013). Cuprous oxide nanoparticles inhibit the growth and metastasis of melanoma by targeting mitochondria. Cell Death Dis.

[160] Sun T, Yan Y, Zhao Y, Guo F, Jiang C (2012). Copper oxide nanoparticles induce autophagic cell death in a549 cells. PLoS One.

[161] Abudayyak M, Guzel EE, Özhan G (2016). Copper (II) oxide nanoparticles induced nephrotoxicity in vitro conditions. Appl Vitro Toxicol.

[162] Xia L, Wang Y, Chen Y, Yan J, Hao F, Su X, Zhang C, Xu M (2017). Cuprous oxide nanoparticles inhibit the growth of cervical carcinoma by inducing autophagy. Oncotarget.

[163] Laha D, Pramanik A, Maity J, Mukherjee A, Pramanik P, Laskar A, Karmakar P (2014). Interplay between autophagy and apoptosis mediated by copper oxide nanoparticles in human breast cancer cells MCF7. Biochim Biophys Acta.

[164] Roggers R, Kanvinde S, Boonsith S, Oupický D (2014). The practicality of mesoporous silica nanoparticles as drug delivery devices and progress toward this goal. AAPS PharmSciTech.

[165] Napierska D, Thomassen LCJ, Rabolli V, Lison D, Gonzalez L, Kirsch-Volders M, Martens JA, Hoet PH (2009). Size-dependent cytotoxicity of monodisperse silica nanoparticles in human endothelial cells. Small.

[166] Thomassen LCJ, Aerts A, Rabolli V, Lison D, Gonzalez L, Kirsch-Volders M, Napierska D, Hoet PH, Kirschhock CEA, Martens JA (2010). Synthesis and characterization of stable monodisperse silica nanoparticle sols for in vitro cytotoxicity testing. Langmuir.

[167] Ha SW, Neale Weitzmann M, Beck GR (2014). Bioactive silica nanoparticles promote osteoblast differentiation through stimulation of autophagy and direct association with LC3 and p62. ACS Nano.

[168] Wang J, Li Y, Duan J, Yang M, Yu Y, Feng L, Yang X, Zhou X, Zhao Z, Sun Z (2018). Silica nanoparticles induce autophagosome accumulation via activation of the EIF2AK3 and ATF6 UPR pathways in hepatocytes. Autophagy.

[169] Krętowski R, Kusaczuk M, Naumowicz M, Kotyńska J, Szynaka B, Cechowska-Pasko M (2017). The effects of silica nanoparticles on apoptosis and autophagy of glioblastoma cell lines. Nanomaterials (Basel, Switzerland).

[170] Schütz I, Lopez-Hernandez T, Gao Q, Puchkov D, JaBerlinbs S, Nordmeyer D, Schmudde M, Rühl E, Graf CM, Haucke V (2016). Lysosomal dysfunction caused by cellular accumulation of silica nanoparticles. J Biol Chem.

[171] Pool H, Campos-Vega R, Herrera-Hernández MG, García-Solis P, García-Gasca T, Sánchez IC, Luna-Bárcenas G, Vergara-Castañeda H (2018). Development of genistein-PEGylated silica hybrid nanomaterials with enhanced antioxidant and antiproliferative properties on HT29 human colon cancer cells. Am J Transl Res.

[172] Wang J, Yu Y, Lu K, Yang M, Li Y, Zhou X, Sun Z (2017). Silica nanoparticles induce autophagy dysfunction via lysosomal impairment and inhibition of autophagosome degradation in hepatocytes. Int J Nanomed.

[173] Yu Y, Duan J, Yu Y, Li Y, Liu X, Zhou X, Fai Ho K, Tian L, Sun Z (2014). Silica nanoparticles induce autophagy and autophagic cell death in HepG2 cells triggered by reactive oxygen species. J Hazard Mater.

[174] Wei F, Wang Y, Luo Z, Li Y, Duan Y (2017). New findings of silica nanoparticles induced ER autophagy in human colon cancer cell. Sci Rep.

[175] Lipatova Z, Segev N (2015). A role for macro-ER-phagy in ER quality control. PLoS Genet.

[176] Lipatova Z, Shah AH, Kim JJ, Mulholland JW, Segev N (2013). Regulation of ER-phagy by a Ypt/Rab GTPase module. Mol Biol Cell.

[177] Schubert D, Dargusch R, Raitano J, Chan SW (2006). Cerium and yttrium oxide nanoparticles are neuroprotective. Biochem Biophys Res Commun.

[178] Das M, Patil S, Bhargava N, Kang JF, Riedel LM, Seal S, Hickman JJ (2007). Auto-catalytic ceria nanoparticles offer neuroprotection to adult rat spinal cord neurons. Biomaterials.

[179] Song W, Soo Lee S, Savini M, Popp L, Colvin VL, Segatori L (2014). Ceria nanoparticles stabilized by organic surface coatings activate the lysosome-autophagy system and enhance autophagic clearance. ACS Nano.

[180] Wei PF, Zhang L, Nethi SK, Barui AK, Lin J, Zhou W, Shen Y, Man N, Zhang YJ, Xu J, Patra CR, Wen LP (2014). Accelerating the clearance of mutant huntingtin protein aggregates through autophagy induction by europium hydroxide nanorods. Biomaterials.

[181] Bibee KP, Cheng YJ, Ching JK, Marsh JN, Li AJ, Keeling RM, Connolly AM, Golumbek PT, Myerson JW, Hu G, Chen J, Shannon WD, Lanza GM, Weihl CC, Wickline SA (2014). Rapamycin nanoparticles target defective autophagy in muscular dystrophy to enhance both strength and cardiac function. FASEB J.

[182] Lin J, Huang Z, Wu H, Zhou W, Jin P, Wei P, Zhang Y, Zheng F, Zhang J, Xu J, Hu Y, Wang Y, Li Y, Gu N, Wen L (2014). Inhibition of autophagy enhances the anticancer activity of silver nanoparticles. Autophagy.

[183] Wu Q, Jin R, Feng T, Liu L, Yang L, Tao Y, Anderson JM, Ai H, Li H (2017). Iron oxide nanoparticles and induced autophagy in human monocytes. Int J Nanomed.

[184] Blanco J, Tomás-Hernández S, García T, Mulero M, Gómez M, Domingo JL, Sánchez DJ (2018). Oral exposure to silver nanoparticles increases oxidative stress markers in the liver of male rats and deregulates the insulin signalling pathway and p53 and cleaved caspase 3 protein expression. Food Chem Toxicol.

[185] Wu H, Lin J, Liu P, Huang Z, Zhao P, Jin H, Ma J, Wen L, Gu N (2016). Reactive oxygen species acts as executor in radiation enhancement and autophagy inducing by AgNPs. Biomaterials.

[186] Shi M, Cheng L, Zhang Z, Liu Z, Mao X (2015). Ferroferric oxide nanoparticles induce prosurvival autophagy in human blood cells by modulating the Beclin 1/Bcl-2/VPs34 complex. Int J Nanomed.

[187] Nowak JS, Mehn D, Nativo P, García CP, Gioria S, Ojea-Jiménez I, Gilliland D, Rossi F (2014). Silica nanoparticle uptake induces survival mechanism in A549 cells by the activation of autophagy but not apoptosis. Toxicol Lett.

[188] Voicu SNP, Dinu D, Sima C, Hermenean A, Ardelean A, Codrici E, Stan MS, Zărnescu O, Dinischiotu A (2015). Silica nanoparticles induce oxidative stress and autophagy but not apoptosis in the MRC-5 cell line. Int J Mol Sci.

[189] Shen TP, Zhu WC, Yang L, Liu L, Jin RR, Duan JM, Anderson JM, Ai H (2018). Lactosylated *N*-alkyl polyethylenimine coated iron oxide nanoparticles induced autophagy in mouse dendritic cells. Regen Biomater.

[190] Liu Y, Yu H, Zhang X, Wang Y, Song Z, Zhao J, Shi H, Li R, Wang Y, Zhang LW (2018). The protective role of autophagy in nephrotoxicity induced by bismuth nanoparticles through AMPK/mTOR pathway. Nanotoxicology.

[191] Lin J, Liu Y, Wu H, Huang Z, Ma J, Guo C, Gao F, Jin P, Wei P, Zhang Y, Liu L, Zhang R, Qiu L, Gu N, Wen L (2018). Key role of TFEB nucleus translocation for silver nanoparticle-induced cytoprotective autophagy. Small.

[192] Duan J, Yu Y, Yu Y, Li Y, Wang J, Geng W, Jiang L, Li Q, Zhou X, Sun Z (2014). Silica nanoparticles induce autophagy and endothelial dysfunction via the PI3K/Akt/mTOR signaling pathway. Int J Nanomed.

[193] Guo C, Yang M, Jing L, Wang J, Yu Y, Li Y, Duan J, Zhou X, Li Y (2016). Amorphous silica nanoparticles trigger vascular endothelial cell injury through apoptosis and autophagy via reactive oxygen species-mediated MAPK/Bcl-2 and PI3K/Akt/mTOR signaling. Int J Nanomed.

[194] Orlando A, Cazzaniga EM, Tringali M, Gullo F, Becchetti A, Minniti S, Taraballi F, Tasciotti E, Re F (2017). Mesoporous silica nanoparticles trigger mitophagy in endothelial cells and perturb neuronal network activity in a size- and time-dependent manner. Int J Nanomed.

[195] Halamoda Kenzaoui B, Chapuis Bernasconi C, Guney-Ayra S, Juillerat-Jeanneret L (2012). Induction of oxidative stress, lysosome activation and autophagy by nanoparticles in human brain-derived endothelial cells. Biochem J.

[196] Kim J-Y, Park J-H, Kim M, Jeong H, Hong J, Chuck RS, Park CY (2017). Safety of nonporous silica nanoparticles in human corneal endothelial cells. Sci Rep.

[197] Li Q, Hu H, Jiang L, Zou Y, Duan J, Sun Z (2016). Cytotoxicity and autophagy dysfunction induced by different sizes of silica particles in human bronchial epithelial BEAS-2B cells. Toxicol Res.

[198] Xie H, Wu J (2016). Silica nanoparticles induce alpha-synuclein induction and aggregation in PC12-cells. Chem Biol Interact.

[199] Zhang L, Wang XQ, Miao YM, Chen ZQ, Qiang PF, Cui LQ, Jing H, Guo YQ (2016). Magnetic ferroferric oxide nanoparticles induce vascular endothelial cell dysfunction and inflammation by disturbing autophagy. J Hazard Mater.

[200] Song M, Zhang R, Dai Y, Gao F, Chi H, Lv G, Chen B, Wang X (2006). The in vitro inhibition of multidrug resistance by combined nanoparticulate titanium dioxide and UV irradition. Biomaterials.

[201] Li Q, Wang X, Lu X, Tian H, Jiang H, Lv G, Guo D, Wu C, Chen B (2009). The incorporation of daunorubicin in cancer cells through the use of titanium dioxide whiskers. Biomaterials.

[202] Chen Y, Wan Y, Wang Y, Zhang H, Jiao Z (2011). Anticancer efficacy enhancement and attenuation of side effects of doxorubicin with titanium dioxide nanoparticles. Int J Nanomed.

[203] Chihara Y, Fujimoto K, Kondo H, Moriwaka Y, Sasahira T, Hirao Y, Kuniyasu H (2007). Anti-tumor effects of liposome-encapsulated titanium dioxide in nude mice. Pathobiology.

[204] Lagopati N, Kitsiou PV, Kontos AI, Venieratos P, Kotsopoulou E, Kontos AG, Dionysiou DD, Pispas S, Tsilibary EC, Falaras P (2010). Photo-induced treatment of breast epithelial cancer cells using nanostructured titanium dioxide solution. J Photochem Photobiol A Chem.

[205] Lai TY, Lee WC (2009). Killing of cancer cell line by photoexcitation of folic acid-modified titanium dioxide nanoparticles. J Photochem Photobiol A Chem.

[206] Popp L, Tran V, Patel R, Segatori L (2018). Autophagic response to cellular exposure to titanium dioxide nanoparticles. Acta Biomater.

[207] Lopes VR, Loitto V, Audinot JN, Bayat N, Gutleb AC, Cristobal S (2016). Dose-dependent autophagic effect of titanium dioxide nanoparticles in human HaCaT cells at non-cytotoxic levels. J Nanobiotechnol.

[208] Wei L, Wang J, Chen A, Liu J, Feng X, Shao L (2017). Involvement of PINK1/parkin-mediated mitophagy in ZnO nanoparticle-induced toxicity in BV-2 cells. Int J Nanomed.

[209] Paris I, Perez-Pastene C, Couve E, Caviedes P, LeDoux S, Segura-Aguilar J (2009). Copper dopamine complex induces mitochondrial autophagy preceding caspase-independent apoptotic cell death. J Biol Chem.

[210] Zhang J, Zou Z, Wang B, Xu G, Wu Q, Zhang Y, Yuan Z, Yang X, Yu C (2018). Lysosomal deposition of copper oxide nanoparticles triggers HUVEC cells death. Biomaterials.

[211] Horejsi V (1998). The leucocyte antigen factsbook, 2nd edn. Immunol Today.

[212] Xu Y, Wang L, Bai R, Zhang T, Chen C (2015). Silver nanoparticles impede phorbol myristate acetate-induced monocyte-macrophage differentiation and autophagy. Nanoscale.

[213] Park EJ, Umh HN, Kim SW, Cho MH, Kim JH, Kim Y (2014). ERK pathway is activated in bare-FeNPs-induced autophagy. Arch Toxicol.

[214] Johnson BM, Fraietta JA, Gracias DT, Hope JL, Stairiker CJ, Patel PR, Mueller YM, McHugh MD, Jablonowski LJ, Wheatley MA, Katsikis PD (2015). Acute exposure to ZnO nanoparticles induces autophagic immune cell death. Nanotoxicology.

[215] Roy R, Singh SK, Chauhan LKS, Das M, Tripathi A, Dwivedi PD (2014). Zinc oxide nanoparticles induce apoptosis by enhancement of autophagy via PI3K/Akt/mTOR inhibition. Toxicol Lett.

[216] Kim S, Jang J, Kim H, Choi H, Lee K, Choi I-H (2012). The effects of silica nanoparticles in macrophage cells. Immune Netw.

[217] Chen L, Liu J, Zhang Y, Zhang G, Kang Y, Chen A (2018). The toxicity of silica nanoparticles to the immune system.

[218] Levine B, Kroemer G (2008). Autophagy in the pathogenesis of disease. Cell.

[219] Lorin S, Hamai A, Mehrpour M, Codogno P (2013). Autophagy regulation and its role in cancer. Semin Cancer Biol.

[220] Chude C, Amaravadi R (2017). Targeting autophagy in cancer: update on clinical trials and novel inhibitors. Int J Mol Sci.

